# Histone lysine dimethyl-demethylase KDM3A controls pathological cardiac hypertrophy and fibrosis

**DOI:** 10.1038/s41467-018-07173-2

**Published:** 2018-12-07

**Authors:** Qing-Jun Zhang, Tram Anh T. Tran, Ming Wang, Mark J. Ranek, Kristen M. Kokkonen-Simon, Jason Gao, Xiang Luo, Wei Tan, Viktoriia Kyrychenko, Lan Liao, Jianming Xu, Joseph A. Hill, Eric N. Olson, David A. Kass, Elisabeth D. Martinez, Zhi-Ping Liu

**Affiliations:** 1 Department of Internal Medicine-Cardiology, UT Southwestern Medical Center, Dallas, TX 75390 USA; 2 Hamon Center for Therapeutic Oncology Research, UT Southwestern Medical Center, Dallas, TX 75390 USA; 3 Nephrology Center of Integrated Traditional Chinese and Western Medicine, Zhujiang Hospital, Southern Medical University, Guangzhou, Guangdong, 510280 P.R. China; 4 Division of Cardiology, Department of Medicine, The Johns Hopkins Medical Institutions, Baltimore, MD 21205 USA; 5 Department of Molecular Biology, UT Southwestern Medical Center, Dallas, TX 75390 USA; 6 Department of Molecular and Cellular Biology and Dan L. Duncan Comprehensive Cancer Center, Baylor College of Medicine, Houston, TX 77030 USA; 7 Department of Pharmacology, UT Southwestern Medical Center, Dallas, TX 77030 USA

## Abstract

Left ventricular hypertrophy (LVH) is a major risk factor for cardiovascular morbidity and mortality. Pathological LVH engages transcriptional programs including reactivation of canonical fetal genes and those inducing fibrosis. Histone lysine demethylases (KDMs) are emerging regulators of transcriptional reprogramming in cancer, though their potential role in abnormal heart growth and fibrosis remains little understood. Here, we investigate gain and loss of function of an H3K9me2 specific demethylase, *Kdm3a*, and show it promotes LVH and fibrosis in response to pressure-overload. Cardiomyocyte KDM3A activates Timp1 transcription with pro-fibrotic activity. By contrast, a pan-KDM inhibitor, JIB-04, suppresses pressure overload-induced LVH and fibrosis. JIB-04 inhibits KDM3A and suppresses the transcription of fibrotic genes that overlap with genes downregulated in *Kdm3a*-KO mice versus WT controls. Our study provides genetic and biochemical evidence for a pro-hypertrophic function of KDM3A and proof-of principle for pharmacological targeting of KDMs as an effective strategy to counter LVH and pathological fibrosis.

## Abstract (web summary)

Histone lysine demethylases (KDMs) can mediate transcriptional reprogramming in disease states. Here the authors show that KDM3A promotes left ventricular hypertrophy and cardiac fibrosis by activating the transcription of Timp1, and that pharmacological inhibition of KDM3A attenuates cardiac remodeling induced by pressure overload.

## Introduction

Heart disease remains a major cause of morbidity and mortality worldwide^1^. Upon exposure to sustained pathological mechanical load or ischemia/reperfusion (I/R) injury, the myocardium undergoes pathological remodeling involving histopathologic, structural, functional, and electro-physiological maladaptations, often coupled with abnormal muscle growth, or ventricular hypertrophy (LVH)^2^. Clinical therapy currently focuses on blocking neurohormonal stimulation and improving hemodynamic load. However, research over the past few decades has dissected signaling pathways as well as genetic and epigenetic factors that govern the remodeling process^2–13^, unveiling novel therapeutic targets.

A hallmark of pathological cardiac remodeling is the reactivation of fetal genes repressed during postnatal development, and dysregulation of genes regulating matrix remodeling, growth, and metabolism. This transcriptional reprogramming can occur through epigenetic modifications, including histone methylation. Histone methylation is the second most conserved posttranslational modification, and controls a multitude of genomic functions, most notably gene transcription. Di- and tri-methylation of histone 3 lysine 9 (H3K9me2 and H3K9me3) are conserved modifications normally associated with transcriptional silencing and are downregulated in hypertrophic and failing hearts in mouse and humans^3,5^. Methylation is dynamically regulated by lysine methyltransferases (KMTs) and lysine demethylases (KDMs). We previously reported that KDM4A/JMJD2A, an H3K9me3 demethylase, promotes pressure overload-induced LVH associated with re-expression of fetal genes and activation of sarcomere protein four and a half limb domains protein 1 (FHL1)^3^. Recently, the H3K9me2 dimethyltransferase EHMT1/2 was found to protect mice against pressure overload-induced LVH with increased global H3K9me2 level^5^. Importantly, multiple models of LVH exhibit upregulation of H3K9 demethylases, including H3K9me2 KDMs, suggesting that maintaining H3K9 methylation may be important to counter LVH. Among the K9me2/me1 demethylases, KDM3A is highly expressed in the heart and has wide transcriptional reprogramming capacity during tissue remodeling, including spermatogenesis and metabolic re-wiring^14–18^. KDM3A is also upregulated in human heart failure patients^19^. However, its role in pathological cardiac remodeling is unknown.

An aspect that makes KDMs particularly intriguing is their association with cancer and neurological disease, which has led to development of targeted inhibitors^20^. We previously generated a series of small molecule KDM inhibitors with varying specificity toward individual KDM members and associated antitumor efficacy^21,22^. Among these, JIB-04 is a pan-inhibitor of KDMs with oral antitumor activity without overt toxicity^22^.

Here, we show that KDM3A promotes LVH, notably inducing fibrosis-related gene expression, and these effects can be suppressed by JIB-04. Using KDM3A-overexpressing *Kdm3a*-Tg and *Kdm3a* knockout (KO) mice, we demonstrate KDM3A is sufficient and necessary to promote LVH in response to pressure overload-induced by trans-aortic constriction (TAC)^16^ surgery. Gene-profiling and gene ontology (GO) analysis indicate that KDM3A particularly controls extracellular matrix biology, triggering fibrosis coupled with *Timp1*^23^, and enhanced extracellular matrix (ECM) gene expression. JIB-04 ameliorates these changes, with transcriptional changes that strongly overlap with those in *Kdm3a-*KO mice.

## Results

### KDM3A promotes cardiomyocyte hypertrophy in vitro

**Fig. 1:**
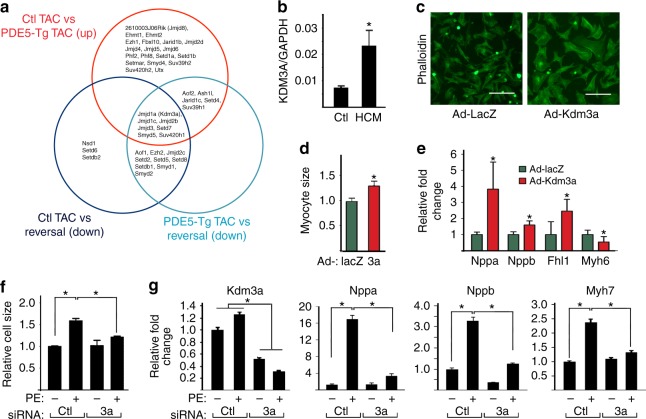
KDM3A promotes cardiomyocyte hypertrophy in vitro. **a** Venn diagram showing differentially expressed genes that are involved in histone methylation and are either up or downregulated in PDE5-Tg versus control littermates, PED5-Tg treated with sildenafil (Reversal) versus control littermates, and reversal versus PDE5-Tg mice after TAC. **b** KDM3A mRNA in human patients with hypertrophic cardiomyopathy (HCM) (*n* = 5) and normal controls. *p *<* *0.05* (*t* test). **c** Immunofluorescence micrographs of NRVMs transduced with adenoviruses expressing either LacZ or Kdm3a. Cells were stained with phalloidin. Scale bar, 100 μm. **d** Relative cell size from cells in **c**. *n* = 3 ± SEM, *p *<* *0.05* (*t* test). **e** Relative fold change of mRNA of gene associated with hypertrophic remodeling in NRVMs transduced with either Ad-LacZ or Ad-Kdm3a (*n* = 3 ± SEM). mRNAs were normalized against internal Gapdh. *p *<* *0.05* (*t* test). **f**–**g** NRVMs were transfected with control siRNA or Kdm3a specific siRNA, treated with or without PE. Cells were fixed and stained with phalloidin for measurement of cell size (**g**) or harvested for measurement of relative mRNA of Kdm3a and fetal gene markers (**g**). mRNA were normalized against internal GAPDH. *n* = 3 ± SEM, *p < 0.05 (ANOVA)

The discovery of KDM3A regulation arose from a series of studies exploring histone epigenetic modifiers that were specifically modulated by successful treatment of mouse models of heart disease^24–26^. We focused on pathways engaging nitric oxide, cyclic GMP, and protein kinase G to benefit overload stress-induced disease, as prior work had shown these each potently reversed pathological hypertrophy, fibrosis, and dysfunction^27^. Microarray analyses were compared with identify change shared by each therapeutic strategy revealing a decline in Kdm3a expression as a common feature (Fig. 1a). KDM3A is also upregulated in human myocardium from patients with hypertrophic heart disease (Fig. 1b), consistent with a previous observation^19^. These data suggested KDM3A may have prohypertrophic effects. To test this, KDM3A was overexpressed in neonatal rat ventricular myocytes (NRVMs) (Supplementary Fig. 1a, b). This increased myocyte size (Fig. 1c, d) and fetal gene expression (*Nppa* & *Nppb*) while reducing adult *Myh6* (Fig. 1e, Supplementary Fig. 1c), are consistent with pathological hypertrophy. Conversely, specific siRNA knockdown of Kdm3a attenuated phenylephrine (PE)-induced hypertrophy (Fig. 1f, Supplementary Fig. 1d–f), reducing *Nppa* and *Nppb* (Fig. 1g).

### KDM3A promotes TAC-induced hypertrophic remodeling in vivo

**Fig. 2:**
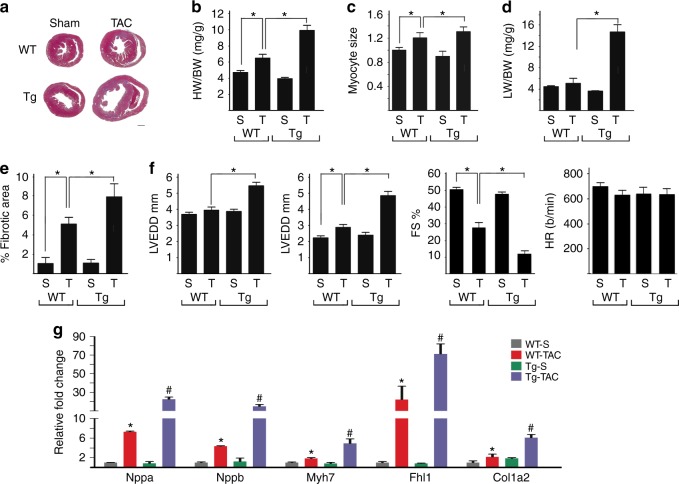
KDM3A promotes TAC-induced hypertrophic remodeling. WT and *Kdm3a*-Tg (Tg) mice were subjected to Sham (S) and TAC (T) surgery. Echocardiograph was performed on mice and hearts were harvested after 6 weeks for histological and biochemical analysis. **a** H&E staining of histologic sections of WT and *Kdm3a*-Tg mouse hearts. Scale bar, 1 mm. **b** HW/BW, **c** relative myocyte cell size, **d** LW/BW, and **e** relative fibrotic area of WT and *Kdm3a*-Tg mouse hearts. **f** Left ventricular end diastolic diameter (LVEDD), left ventricular end systolic diameter (LVESD), percent of fractional shortening (FS%), and heart rate (HR, beat/min). **g** Relative mRNA of canonical fetal gene markers (Nppa, Nppb, and Myh7), Fhl1, and Col1a2. *n* = 6-10 ± SEM. ^*, #^*p* *<* 0.05 (ANOVA). *WT TAC vs. WT Sham. ^#^, Tg-TAC vs. WT-TAC

To test whether KDM3A promotes hypertrophic remodeling in vivo, transgenic mice with postnatal myocyte-selective KDM3A overexpression were generated (Supplementary Fig. 2a, b). Results presented are from line Tg-29. In the absence of stress, *Kdm3a*-Tg mice displayed normal cardiac morphology and function as in WT littermates (data not shown). However, after 6 weeks of TAC, *Kdm3a*-Tg mice showed an exacerbated hypertrophic response (Fig. 2a) over WT littermates with greater heart weight/body weight (HW/BW) ratio (Fig. 2b) and cardiomyocyte size (Fig. 2c). *Kdm3a*-Tg TAC mice had more severe heart failure reflected by greater lung weight (Fig. 2d), more interstitial fibrosis than in WT-TAC (Fig. 2e, Supplementary Fig. 2c), and worse cardiac function (Fig. 2f). As in isolated cells, myocardial fetal and sarcomere gene expression changes reflected worse pathological hypertrophy (Fig. 2g). Similar results were observed in the other transgenic line Tg-24 (Supplementary Fig. 2d).

### *Kdm3a*-deletion protects mice against TAC-induced LVH

**Fig. 3:**
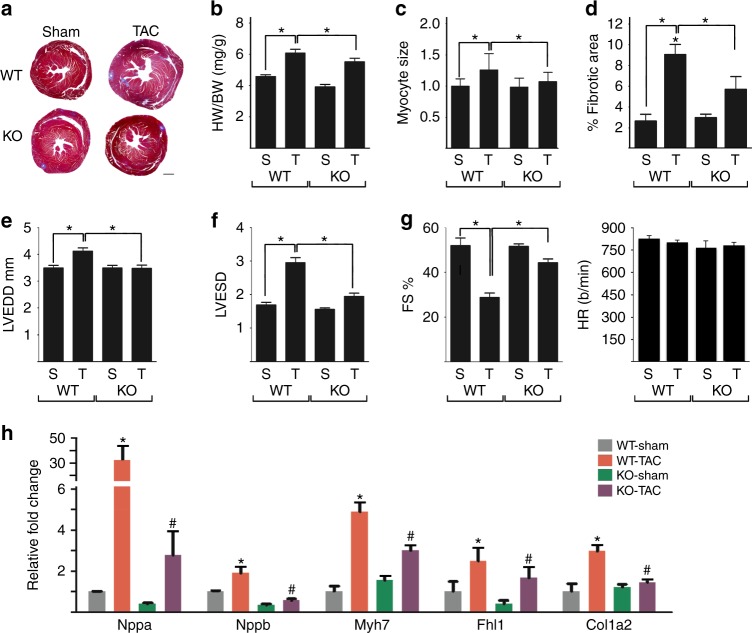
*Kdm3a*-deficiency protects mice again TAC-induced hypertrophic remodeling. WT and *Kdm3a* KO (KO) mice were subjected to Sham and TAC surgery. Hearts were echoed and harvested 6 weeks post-surgery for histological and biochemical analysis. **a** H&E staining of histologic sections of WT and KO mouse hearts. Scale bar, 1 mm. **b** HW/BW, **c** relative myocyte size, and **d** percent of fibrotic area of WT and KO mouse hearts. **e** LVEDD, **f** LVESD, **g** percent FS and heart rate of WT and KO mouse hearts. **h** Relative mRNA of canonical fetal gene markers (Nppa, Nppb, and Myh7), Fhl1, and Col1a2. mRNA transcripts were measured by qRT-PCR, normalized against internal Gapdh, and expressed relative to Sham WT mice. *n* = 6-10 ± SEM. *^, #^*p* < 0.05 (ANOVA). *, WT TAC vs. WT Sham. ^#^, Tg-TAC vs. WT-TAC

Global *Kdm3a*^*-/-*^ (KO) mice generated on a mixed FVB/C57/Bl6J background and littermate controls were subjected to TAC. KO mice are viable with no overt spontaneous cardiac phenotype^28^. Six weeks after TAC, KO hearts were significantly smaller than controls (Fig. 3a), with a lower HW/BW ratio (Fig. 3b), smaller cardiomyocytes (Fig. 3c), less fibrosis (Fig. 3d, Supplementary Fig. 3a), improved cardiac function (Fig. 3e–g), and reduced expression of *Nppa*, *Nppb*, and *Myh7* (Fig. 3h). A separate line of *Kdm3a* KO in C57/Bl6J was generated. These mice had more attenuated hypertrophy in response to TAC (Supplementary Fig. 3b), indicating genetic modifiers of KDM3A-regulated hypertrophic remodeling.

### KDM3A promotes pro-fibrotic gene expression and activates *Timp1*

**Fig. 4:**
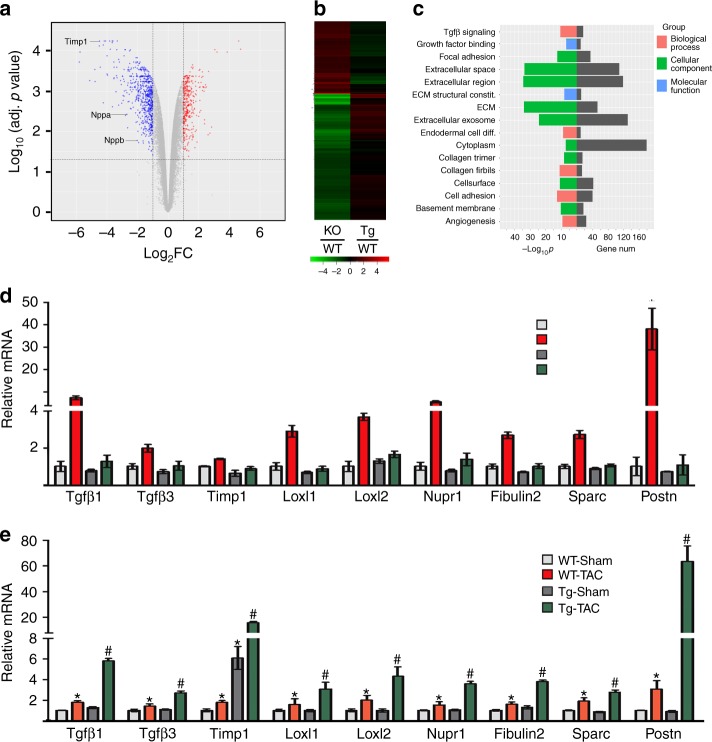
KDM3A promotes transcription of a pro-fibrotic gene program. **a** Volcano plot of differentially expressed genes in *Kdm3a* KO mouse hearts compared to those in WT mouse hearts at 6 weeks post-TAC surgeries. Timp1 is among the most downregulated genes in KO hearts. **b** Heatmaps of differentially expressed genes in KO vs WT and *Kdm3a*-Tg vs WT mouse hearts at 6 weeks post-TAC surgery, showing differentially expressed genes are inversely regulated in KO vs Tg hearts. **c** GO analysis of differentially downregulated genes in KO vs WT TAC mouse hearts. Relative mRNA of genes involved in fibrosis in *Kdm3a*^*-/-*^ (**d**) and *Kdm3a*-Tg (**e**) mouse hearts 6 weeks post-Sham and TAC surgery. mRNA transcripts were measured by qRT-PCR, normalized against internal Gapdh, and expressed relative to Sham WT mice. *n* = 5 ± SEM. *^, #^*p* *<* *0.05* (ANOVA). *, WT TAC vs. WT Sham. ^#^, Tg-TAC vs. WT-TAC

We performed gene-profiling with *Kdm3a* KO and WT littermate hearts at week 6 post Sham or TAC. There are 537 and 300 genes that were downregulated ( > twofold) and upregulated ( > twofold), respectively, in KO versus WT. Prominent changes were in genes involved in extracellular matrix (ECM) remodeling, with *Timp1* being the most downregulated (Fig. 4a). These same genes are more upregulated in *Kdm3a*-Tg over WT TAC (Fig. 4b). Gene ontology (GO) analysis indicated the most differentially downregulated genes in KO TAC involved ECM biology and fibrosis (Fig. 4c) while those upregulated involved energy metabolism (Supplementary Fig. 4a). This is very similar to gene-modulated changes observed from PKG activation by sildenafil (PDE5 inhibitor) in the same TAC model^25^. Differential gene expression was confirmed by qRT-PCR in independent samples (Fig. 4d, e). In addition to pro-fibrotic genes that were upregulated in TAC *Kdm3a*-Tg heart versus WT (Supplementary Fig. 4b), we found that Adam9 and Adam19 that encode ECM-degrading proteins were also upregulated in TAC *Kdm3a*-Tg vs WT (Supplementary Fig. 4c). Consistent with upregulation of a fibrotic gene program in *Kdm3a*-Tg TAC mouse hearts, we also detected upregulation of the Tgfβ1 and Tgfβ1-activated signaling pathway by western blot analysis (Supplementary Fig. 4d).

**Fig. 5:**
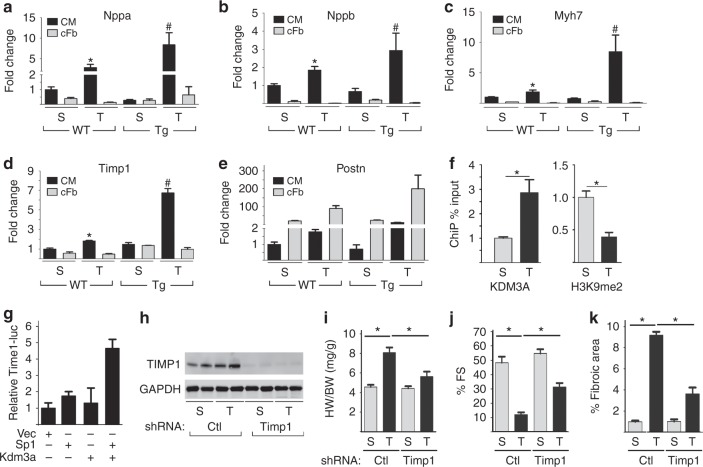
KDM3A activates the transcription of Timp1 in cardiomyocytes. Cardiomyocytes (CM) and cardiac fibroblasts (cFb) were isolated from WT and Kdm3a-Tg mouse hearts at week 6 post-Sham and TAC surgery. **a**–**e** Transcripts of fetal genes and fibrosis-related ECM genes were measured by qRT-PCR, normalized against internal Gapdh, and expressed relative to WT Sham CM. n = 3 ± SEM, *^, #^*p* *<* 0.05 by student *t* test. *, WT TAC vs WT Sham. ^#^, Tg-TAC vs WT-TAC. **f** Cardiomyocytes from *Kdm3a*-Tg mouse hearts at week 6 post-Sham and TAC surgery were isolated and used for ChIP assay with antibodies against KDM3A (left panel) or H3K9me2 (right panel). The relative occupancies of KDM3A and H3K9me2 at Timp1 promoter were normalized against Input and expressed relative to Sham control. n = 3 ± SEM, *p *<* *0.05* (*t* test). **g** Timp1-Luciferase reporter was transfected in combination with plasmids as indicate into 293 T cells. Cells were harvested 48 h after transfection. Luciferase activities were normalized against co-transfected β-galactosidase, expressed relative to vector-transfected cells. *n* = 3 ± SEM. (h-k) *Kdm3a-*Tg mice were transduced with adenovirus AAV9 expressing control or Timp1 shRNA and subjected to Sham or TAC surgery. Hearts were echoed and harvested 5 weeks post-surgery. **h** WB of TIMP1 and GAPDH, **i** HW/BW, **j** %FS, and **k** percent of fibrotic area of Sham (S), TAC (T) Kdm3a-Tg mice transduced with control or Timp1 shRNA. *n* = 7 ± SEM, *p *<* 0.05 (ANOVA)

To identify potential direct targets of KDM3A, we isolated adult cardiomyocytes from WT and *Kdm3a*-Tg sham and TAC hearts, and performed microarray experiments. Since KDM3A in *Kdm3a*-Tg mouse hearts is only overexpressed in cardiomyocytes, we reasoned that genes altered in cardiomyocytes of *Kdm3a*-Tg hearts would most likely be direct transcriptional targets. Most differentially expressed ECM genes in *Kdm3a*-Tg hearts proved to be from non-cardiomyocytes (Supplementary Fig. 5a). However, we did find *Timp1* among those upregulated in cardiomyocytes and confirmed its expression as well as other fetal genes using qRT-PCR (Fig. 5a–e). *Timp1* showed a non-significant trend of upregulation in cardiomyocytes of *Kdm3a*-Tg at baseline, and rose significantly more after TAC than in WT cells (Fig. 5d). *Timp1* in non-cardiomyocyte fractions of *Kdm3a*-Tg hearts remained similar to WT hearts, under both sham and TAC conditions (Fig. 5d). Upregulation of TIMP1 at the protein level in *Kdm3a*-Tg mouse hearts (Supplementary Fig. 5b) and in cardiomyocytes (Supplementary Fig. 5c) after TAC was confirmed by western blot analysis. KDM3A-ChIP qPCR in isolated cardiomyocytes indicated that KDM3A can bind to the *Timp1* promoter specifically in response to TAC and binding of KDM3A is associated with downregulation of H3K9me2 (Fig. 5f). Similar results were also observed in the whole heart (Supplementary Fig. 5d). KDM3A is also able to activate a *Timp1*-promoter-driven luciferase reporter (Fig. 5g). We further confirmed that KDM3A is sufficient to activate endogenous *Timp1* transcription in NRVMs (Supplementary Fig. 1c). Taken together, these data suggest that *Timp1* is a direct transcriptional target of KDM3A in the heart.

Timp1 is a secreted ECM protein that has both MMP-dependent^29,30^ and independent functions^23^. Timp1 has been shown to promote association of CD63 and integrin β1 on cardiac fibroblasts, promoting fibrotic gene expression^23^. We speculated that Timp1 from cardiomyocytes may mediate the prohypertrophic and pro-fibrotic function of KDM3A. To test this hypothesis, we knocked down Timp1 in cardiomyocytes using AAV9 adenovirus expressing Timp1 specific shRNA (Fig. 5h, Supplementary Fig. 5e) since AAV9 preferentially transduces cardiomyocytes^31^. Timp1 knockdown in cardiomyocytes of *Kdma3a*-Tg mice attenuated the TAC-induced hypertrophic remodeling and fibrosis (Fig. 5i–k, Supplementary Fig. 5f), suggesting Timp1 indeed mediates the prohypertrophic/fibrotic function of KDM3A in cardiomyocytes.

### JIB-04 blocks TAC-induced LVH and fibrosis

**Fig. 6:**
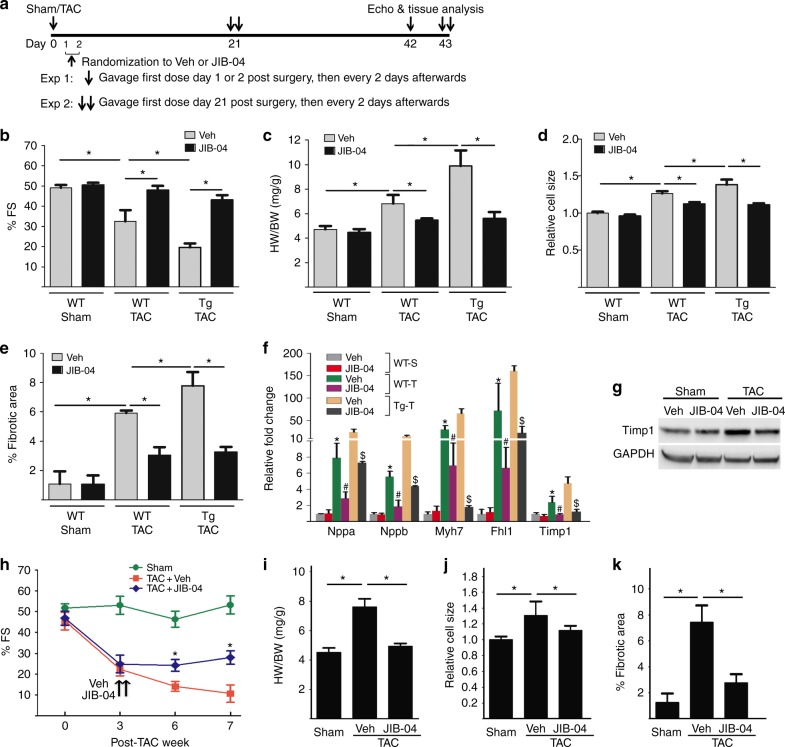
JIB-04 blocks TAC-induced hypertrophic remodeling and fibrosis. **a** Experimental protocol of JIB-04 and control vehicle (Veh) treatment of WT and *Kdm3a*-Tg mice. JIB-04/Veh were given to mice at either day 1 or 2 (exp 1) or 21 (exp 2) post-TAC. **b** %FS, **c** HW/BW ratio, **d** relative cell size, **e** percentage of fibrotic area, and **f** relative fold change of fetal genes and Timp1 of WT sham, WT TAC and *Kdm3a*-Tg TAC mouse heart at the end of experiment 1. *n* = 5-6 ± SEM, *, ^#^, ^$^, p < 0.05 (ANOVA). *, WT TAC vs WT Sham. ^#^, JIB-04 WT TAC vs Veh WT-TAC. ^$^, JIB-04 Tg TAC vs Veh TG TAC. **g** Western blot of TIMP1 in vehicle or JIB-04 treated WT mouse hearts at post-sham/TAC week 6 in experiment 1. **h** %FS over treatment period, **i** HW/BW, **j** relative cell size, and **k** % fibrotic area at end of experiment 2 of Sham and TAC *Kdm3a*-Tg mouse hearts treated with vehicle or JIB-04. *n* = 5 ± SEM, *p *<* 0.05 (ANOVA)

We have identified small molecule inhibitors of KDMs including JIB-04 that showed oral activity in tumor suppression^21,22^ and blocked KDM3A in vitro. JIB-04 inhibited PE-induced NRVM hypertrophy (Supplementary Fig. 6a), and suppressed KDM3A-promoted hypertrophy of NRVMs in vitro (Supplementary Fig. 6b). To test if JIB-04 targets KDM3A in TAC-induced pathological hypertrophic remodeling in vivo, we treated sham and TAC WT and *Kdm3a*-Tg mice with JIB-04 or vehicle (administered by gavage) (Fig. 6a) at a dose with no overt toxicity^22^. Administration of JIB-04 preserved cardiac function (Fig. 6b) and suppressed hallmarks of pathological hypertrophic remodeling in both WT and *Kdm3a*-Tg hearts, including attenuated HW/BW (Fig. 6c) and reductions in cardiomyocyte size (Fig. 6d), fibrosis (Fig. 6e), and canonical fetal gene expression (Fig. 6f). JIB-04 also attenuated upregulation of TAC-induced Timp1 expression at RNA (Fig. 6f) and protein levels (Fig. 6g). We further tested if JIB-04 could prevent or reverse the progression of an already established disease by administering JIB-04 to *Kdm3a*-Tg mice 21 days after TAC surgery (Fig. 6a) when the cardiac dysfunction became evident (Fig. 6h). JIB-04 halted the disease progression with attenuated hypertrophic remodeling and fibrosis compared to vehicle treatment (Fig. 6h–k, Supplementary Fig. 6c).

We compared the efficacy of JIB-04 in blocking TAC-induced LVH with that of lysyl oxidase (Lox) inhibitor β-aminopropionitrile (BAPN). Lox catalyzes collagen cross-linking. Inhibition of Lox with BAPN attenuates volume overload-induced pathological ECM remodeling in rat hearts^32,33^ and in angiotensin II-induced vascular stiffness in mice^34^. Administration of BAPN post-TAC surgery suppressed LVH and fibrosis of *Kdm3a*-Tg mice to the WT TAC level, including cardiac function and gravimetric measurements (Supplementary Fig. 6d–g). However, unlike JIB-04, BAPN did not further reduce the adverse remodeling beyond the WT TAC level. These results suggest that JIB-04 is a more effective agent; not only in ameliorating KDM3A-activated fibrosis but also for TAC-induced cardiac hypertrophy.

### JIB-04 protects mice against ischemia/reperfusion (I/R) injury

**Fig. 7:**
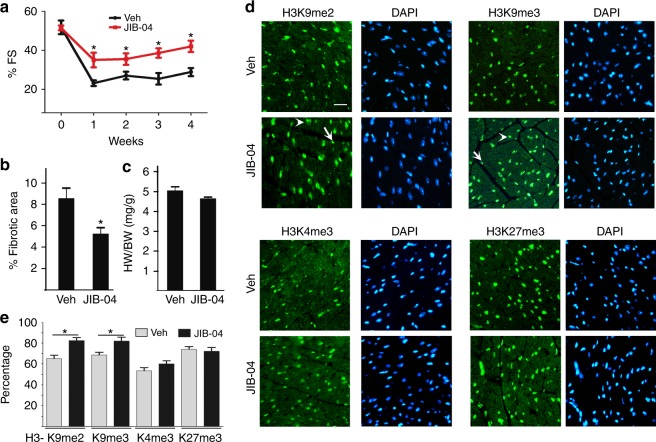
JIB-04 protects mice against I/R injury. WT mice were subjected to I/R surgery and given vehicle (Veh) or JIB-04 as shown in Fig. 6a. Echocardiographs were performed weekly for 4 weeks to measure the fractional shortening (**a**). **b** Percent fibrotic area and **c** HW/BW ratio of mice treated with Veh or JIB-04 at 4 weeks post-I/R surgery. **d** Immunofluorescences of H3K9me2, H3K9me3, H3K4me3, and H3K27me3 of hearts treated with Veh or JIB-04 at week 4 post-I/R surgery. H3K9me2/3 in both cardiomyocyte (arrow head) and non-cardiomyocytes (arrow) were affected by JIB-04 treatment. Scale bar, 20 μm. **e** Quantification of percent of cells in **d** stained with methylated histones as indicated. Staining of methylated histones was normalized against Dapi stained cells. *p *<* 0.05, *n* = 5 ± SEM (ANOVA)

Myocardial fibrosis in TAC-induced hypertrophic remodeling is often considered as a reactive fibrosis. To test whether JIB-04 can repress reparative fibrosis that is mediated by inflammatory influx and activated myofibroblasts, the I/R injury model was chosen to limit the extent of injury to mainly fibrosis. I/R injured WT mice were treated with either vehicle or JIB-04 following a similar protocol as described in Fig. 6a. I/R injury reduced cardiac function and increased fibrosis, both partially blocked by JIB-04 treatment as compared with vehicle treatment (Fig. 7a, b). JIB-04 treatment did not significantly alter HW/BW (Fig. 7c). Immunofluorescence staining of methylated histone lysine showed upregulation of H3K9me2 and H3K9me3 in JIB-04-treated mouse hearts compared with vehicle-treated controls (Fig. 7d, e), suggesting JIB-04 may target similar KDMs in a mouse I/R injury model as in TAC. Changes of H3K9me3/me2 were seen in both cardiomyocytes (arrow head) and non-cardiomyocytes (arrow, likely fibroblasts). JIB-04 did not change levels of H3K4me3 or H3K27me3.

### JIB-04 targets H3K9me2 and genes involved in myocardial fibrosis

**Fig. 8:**
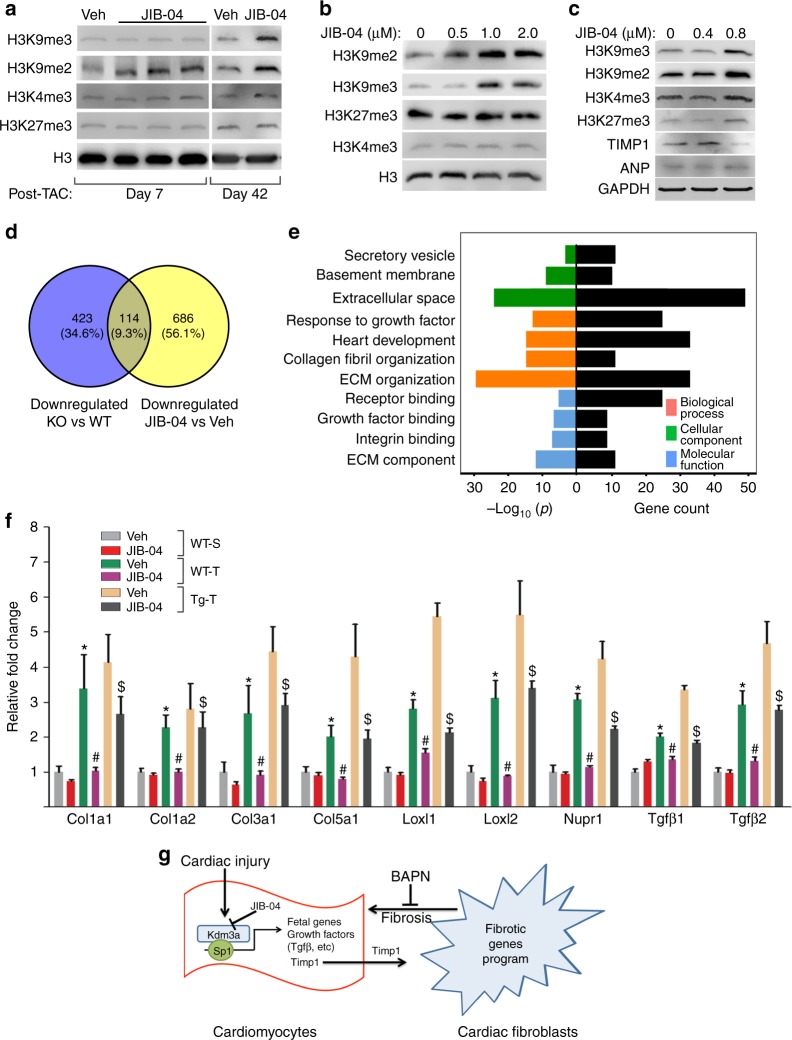
JIB-04 targets H3K9me2 demethylase and fibrotic gene program. **a** Western blots of indicated proteins in lysates from WT mouse hearts treated with vehicle (Veh) or JIB-04 harvested at day 7 and day 42 post-TAC surgeries. **b**, **c** Western blots of indicated proteins from lysates of NRVMs (**b**) and human iPSC-CM (**c**) treated with various concentration of JIB-04. **d** Venn diagram showing overlapping of downregulated gene sets between KO vs WT and JIB-04 vs Veh treated mice. **e** GO analysis of 114 overlapping genes in **d**, showing significant enrichment of gene set in ECM and growth factor/integrin signaling. **f** qRT-PCR of representative genes in **d**. *n* = 3 ± SEM, *, ^#^, ^$^, p < 0.05 (ANOVA). *, WT TAC vs WT Sham. ^#^, JIB-04 WT TAC vs Veh WT TAC. ^$^, JIB-04 Tg TAC vs Veh TG TAC. **g** Schematics showing potential mechanism of action for KDM3A and inhibitor JIB-04

To identify the potential targets of JIB-04 in the heart, we measured the levels of methylated histone lysines by western blot in WT sham and TAC mice with or without JIB-04 treatment. JIB-04 treatment of TAC mouse hearts resulted in upregulation of H3K9me2 with little change in H3K4me3 and H3K27me3 (Fig. 8a). Change of global H3K9me3 was also seen at a later post-TAC time point. JIB-04 treatment of NRVMs (Fig. 8b) and human induced pluripotent stem cell derived cardiomyocytes (hiPSC-CM) (Fig. 8c) also resulted in upregulation of H3K9me2 and H3K9me3. These results suggested that JIB-04 targets mainly the H3K9me2 and H3K9me3 demethylases in the heart.

We performed RNA-seq of vehicle and JIB-04-treated sham and TAC WT mice to identify potential transcriptional targets of JIB-04. JIB-04 treatment of sham WT mice resulted in 800 differentially expressed genes ( < or > twofold) in the heart compared with vehicle-treated sham mice. GO analysis did not show any significantly enriched biological processes. JIB-04 treatment of TAC mice resulted in 800 downregulated ( < twofold) and 574 upregulated genes ( > twofold) compared with vehicle-treated TAC mice. We compared downregulated genes in JIB-04 versus vehicle-treated TAC mice with those downregulated in *Kdm3a*-KO versus WT TAC mice. There were 114 overlapping genes between the two sets (Fig. 8d). GO analysis indicated that they are predominantly ECM proteins and involved in fibrosis and growth factor/integrin signaling (Fig. 8e). The genes include fetal genes associated with hypertrophic growth, the gene encoding sarcomere protein FHL1, and KDM3A-regulated fibrotic genes such as *Timp1* (Figs 6f, 8f) as expected.

## Discussion

In this study, we reveal a previously unidentified role of KDM3A in promoting pathological ventricular remodeling, and corresponding anti-cardiac hypertrophy/remodeling efficacy of the KDM inhibitor JIB-04. KMTs and KDMs are emerging central regulators of pathological cardiac hypertrophy and failure^4^. While KDMs are already established as drug targets for cancer and neurological diseases, to our knowledge, the current study shows for the first time that KDM inhibitors can ameliorate forms of heart disease.

Our prior study found the H3K9me3 demethylase KDM4A promotes LVH and activates transcription of associated fetal genes and the gene encoding sarcomere protein FHL1^3^. Here, we demonstrate the H3K9me2 demethylase KDM3A has similar effects on pathological remodeling, but does so with targeted regulation of extracellular matrix-*Timp1* and TGFβ associated genes. Liu et al. showed that KDM7B/PHF8 is also expressed in the heart and represses TAC-induced LVH remodeling^35^. KDM7B demethylates di- and mono-methylated histone lysines (H3K9me1/2, H3K27me2, and H4K20me1) although its activity on H3K9me2 is dependent on its PHD domain interacting with active H3K4me3 marks at target genes^36,37^. KDM3A lacks PHD domains and robustly demethylates H3K9me2 substrates. Since KDM7B has been shown to regulate expression of genes involved in cell cycle progression^38^, rRNA^39^, and Akt/mTOR signaling pathways^35^ in the heart, it will be of future interest to determine if these two KDMs have overlapping cardiac substrates. Nonetheless, based on the published data and ours, we speculate that these KDMs regulate distinct sets of target genes.

We identified *Timp1* in cardiomyocytes as a potential target of KDM3A that mediates its pro-fibrotic function since: (1) *Timp1* is upregulated in cardiomyocytes in *Kdm3a*-Tg hearts upon TAC, (2) KDM3A binds the *Timp1*-promoter after TAC and promotes transcription as assayed by a *Timp1*-promoter-driven luciferase reporter assay, (3) cardiomyocyte *Timp1* knockdown attenuated the post-TAC cardiac remodeling of Kdm3a-Tg mice (Figs. 5), and (4) the KDM3A inhibitor JIB-04 represses hypertrophic remodeling and downregulates the expression of *Timp1* (Fig. 6). TIMP1 has been shown to be important in maintaining the homeostatic balance of myocardial ECM via inhibition of MMPs as well as MMP-independent mechanisms^23,29,30,40,41^. Elevated plasma TIMP1 levels have been associated with myocardial fibrosis and used as a fibrosis biomarker in patients with heart disease^42–44^. Consistently, we observed upregulation of *Timp1* in cardiomyocytes of *Kdm3a*-Tg mice as well as myocardial fibrosis in response to TAC, with most of the fibrotic genes being upregulated in cardiac fibroblasts. Based on previous results established in the literature and our own studies here, it is thus reasonable to speculate that TIMP1 may mediate the pro-fibrotic function of KDM3A. KDM3A-activated TIMP1 in cardiomyocytes may be secreted into the ECM, subsequently activating resident cardiac fibroblasts and leading to myocardial fibrosis (Fig. 8g). That being said, it is possible that KDM3A may have a role in other heart cell types as well as non-histone related functions, which is worthy exploring in the future.

KDM3A was originally identified as a potential epigenetic enzyme that mediates the prohypertrophic function of PDE5 (Fig. 1a). The stress-induced cardiac phenotypes and worsening of fibrotic-associated genes observed with *Kdm3a*-Tg mice are similar to those in models where PDE5 was overexpressed only in cardiomyocytes. Moreover, this is reversed by enhancing PKG activity using PDE5 inhibitors^24^. A mouse model combining inducible PDE5-Tg with the *Kdm3a*-KO could be used to further test for a mechanistic dependence of PKG modulation on KDM3A gene regulation.

Myocardial fibrosis is often considered as a secondary effect of pathological remodeling due to changes in the structure and mechanical properties of the myocardium. Our studies suggest that the pro-fibrotic function of KDM3A may have a causative role in promoting LVH as treatment of post-TAC *Kdm3a*-Tg mice with the fibrosis inhibitor BAPN blocked the development of exacerbated LVH due to transgene expression (Supplementary Fig. 6). This is consistent with the observation that fibrosis was found to precede cardiac hypertrophy in many HCM patients^45^. Pathogenic mutations in sarcomere protein αMHC (Arg403Gln or Arg719Trp) from HCM patients were shown to promote proliferation and fibrotic gene expression of non-cardiomyocytes in pre-hypertrophic *αMHC*^*403/+*^ and *αMHC*^*719/+*^ mouse hearts^46^. We observed similar crosstalk between cardiomyocytes and non-cardiomyocytes in our studies, overexpression of KDM3A in cardiomyocytes promoted expression of fibrotic genes in non-cardiomyocytes (Fig. 5). Moreover, our studies suggest that TIMP1 secreted from cardiomyocytes might mediate this crosstalk in a paracrine fashion. KDM3A is upregulated in human HCM patients (Fig. 1b) and thus may mediate the fibrotic effect of pathogenic mutations of αMHC. This possibility could be tested by *Kdm3a*-deletion or inhibition via JIB-04 in *αMHC*^*403/+*^ and *αMHC*^*719/+*^ mouse hearts. A positive outcome of such testing could provide a new strategy for limiting HCM pathophysiology by preemptively inhibiting KDM3A in HCM patients with sarcomere protein gene mutations and elevated KDM3A expression.

Our data indicated that JIB-04 is effective in suppression of both reactive and reparative fibrosis. The mechanism by which JIB-04 inhibits reparative fibrosis in the I/R model remains to be determined. Myofibroblasts are the major cell type responsible for fibrosis in I/R injury^7,47,48^. JIB-04 treatment of hearts resulted in alteration of H3K9me2 and H3K9me3 in fibroblasts as well as in cardiomyocytes (Fig. 8d); thus, JIB-04 likely has an effect on fibroblast activation and differentiation, which could be tested in the future.

Heart failure and cancer are two of the world’s leading causes of morbidity and mortality, and emerging evidence supports they share common mechanisms at the level of chromatin regulation^10^. Our studies suggest that KDMs of the Jumonji enzyme family represent one such shared mechanism. JIB-04 was identified originally as an antitumor agent^21,22^. However, the current work shows it suppresses pressure overload-induced pathological LVH and myocardial fibrosis. JIB-04 is a pan-KDM inhibitor that inhibits demethylase activities of KDM5 (H3K4me3 demethylase) and KDM6 (H3K27me3 demethylase) in addition to KDM3 and KDM4 in vitro^22^. In NRVMs, JIB04-04 inhibited KDM3 activity at relatively low concentration and KDM4 activity at higher concentration, and had little effect on KDM5 and KDM6 activity at the concentration tested. In heart tissue, we observed changes in global H3K9me2 levels at an early time point of JIB-04 treatment and changes of global H3K9me3 and H3K4me3 level at a later treatment time point. These results suggest that KDM3 may play a more important role in pathogenesis of LVH and be more susceptible to JIB-04 inhibition. KDM3A is upregulated in human HCM and our data showed that JIB-04 is effective in inhibition of KDM3A in human heart cells (Fig. 8c), suggesting that JIB-04 may be a useful therapeutic agent in combating human heart diseases.

In summary, we have established a novel role for KDM3A in LVH and demonstrated that KDM3A promotes LVH and myocardial fibrosis in response to pressure overload. Furthermore, our proof-of-principle studies demonstrated that pharmacologically targeting KDMs might be a valid therapeutic strategy to combat LVH and fibrosis in various heart pathologies.

## Methods

### General mouse management, *Kdm3a*-Tg and *Kdm3a* KO mouse lines

All animal experiments were approved by the Institutional Animal Care and Use Committee (IACUC) of UT Southwestern and performed in adherence to the relevant ethical regulations. Mouse Kdm3a cDNA with 5′ Flag tag was subcloned downstream of αMHC promoter^3^. After removal of plasmid backbone, αMHC-FLAG-KDM3A construct was used to generate transgenic mouse lines at core facility of UT Southwestern Medical Center. Five lines of positive transgenic mouse were genotyped and two lines were used for further experiments. These two lines of transgenic mice show a similar phenotype and the primary data presented are from line 29. *Kdm3a*^+/-^ mouse line was a kind gift of Dr. J Xu^28^.

### TAC and I/R surgery, echocardiography, and histological analysis

Male mice with body weight of 20 to 25 g (age of 6–8 weeks) of various genetic backgrounds as indicated were used. TAC surgery was performed by banding of aortic arc with 7-0 suture together with 27 G syringe needle. The removal of needle results in partial constriction of aorta^3^. For I/R surgeries, the left coronary artery was ligated for 45 min before releasing. Animals were killed after 6 weeks, and histological analyses were performed on fixed heart tissues. Sham-operated animals were used as controls and underwent the same surgical procedure, except banding of suture. Echocardiography was performed on Vevo 2100 (VisualSonics) under conscious, un-sedated condition at specified time points. Mice were killed at time points as indicated. For histological and immunofluorescence analysis, hearts were removed quickly, weighed and fixed with 4% formaldehyde for paraffin embedding and sectioning. H&E and Trichrome staining were performed by the histological core of UT Southwestern following standard protocols. Cardiomyocyte and fibrosis area were quantified with Image J (NIH) on H&E and Trichrome stained sections. For biochemical analysis, hearts were snap frozen in liquid nitrogen within 30 s of removal and stored at −80 °C.

### Drug preparation and administration

JIB-04 was administered by gavage with a dosage of 50 mg/kg. Drug is first dissolved in 12.5% DMSO, then 12.5% cremophor, and thoroughly mixed by vortexing. Right before administration, the drug solution was mixed with water in 3:1 ratio and delivered immediately by gavage.

### RNA purification, qRT-PCR, microarray, and RNA-seq

Total RNA was purified from tissue or cultured cell with TRIZol (Invitrogen) following manufacturer’s protocol. One to five micrograms of total RNA was used for reverse transcription with SSTIII kit (Invitrogen) to generate cDNA. The cDNA is used in SYBR-based real-time qRT-PCR. The sequences of the primers for each gene detected are listed in Supplementary Table 1. Five hundred nanograms of total RNA was used for microarray analysis or RNA-seq with Illumina platform. Both microarray and RNA-seq were carried out by the genomic core facility of UT Southwestern. All microarray and RNA-Seq data were deposited into the NCBI GEO repository (GSE).

### Protein extraction, SDS-PAGE, western blot, and ChIP assay

Proteins were extracted from mouse hearts with T-PER tissue protein extraction reagent (Thermo Scientific) plus proteinase inhibitors. RIPA buffer plus proteinase inhibitors was used to extract protein from cardiomyocytes and cardiac fibroblasts isolated from adult mouse hearts and in vitro cultured NRVMs. Protein samples were quantified and separated with SDS-PAGE before transfer to a nitrocellulose membrane, followed by antibody probing. ChIP assays were performed as described previously^3^ with specific gene primers as indicated (listed in Supplementary Table 1). The following primary antibodies were used: GAPDH (Santa Cruz, sc-20358), H3K9Me2 (Abcam, ab1220), H3K9me3 (Abcam, ab8898), H3K4me3(Active Motif, 39159), and H3K27me3 (Millpore, 07-449), Histone H3(CellSignaling, 4499), FLAG (Sigma-Aldrich, F1804), TIMP1 (Invitrogen, MA1-773), ANP (Novus, NBP2-14873), TGFbeta1 (Abcam, ab-64715), pSMAD3 (Santa Cruz, sc11769), SMAD3(Santa Cruz, sc-133098). All antibodies were diluted 1:1000 in western blot. The original un-cropped western blots with molecular weight markers were presented in supplementary figure 7.

### Phalloidin and immunofluorescence staining

Phalloidin-FITC staining of NRVMs was performed by following the protocol described^49^. Briefly, cells fixed with 4% formaldehyde for 5 min followed by Phalloidin-FITC (1:200) for 10 min at room temperature. Cell sizes were quantified using Image J (NIH). Immunofluorescence staining of NRVMs and paraffin-embedded heart sections was carried out by following standard protocols. Briefly, heart sections were incubated at 60 °C for 30 min, followed by deparaffinization. Antigen retrieval was performed in 1X Antigen Retrieval Citra Solution (BioGenex) with microwave oven. Sections were blocked with 1% BSA in PBS for 1 h at RT, followed by primary antibody and FITC conjugated secondary antibody. Nuclei were stained with DAPI. Finally the slides were mounted with anti-fading mounting buffer (Sigma-Aldrich).

### Preparation of neonatal and adult heart cells

In total, 1–2-day-old neonates from timed-pregnant Sprague Dawley rat (Charles River) were used to separate neonatal rat ventricle myocyte **(**NRVMs) as described^50^. Adult mouse cardiomyocytes and cardiac fibroblasts were isolated from Sham and TAC hearts by the Langendorff method as described^51^. Mouse heart was removed quickly and put to enzyme solution infusion for 20 min. Then heart ventricle tissue was pippetted to release cells. Cardiomyoytes and non-myocytes were separated by repeated low speed centrifuge.

### Adenovirus infection and siRNA transfection

Adenovirus packaging was carried out with AdEasy XL Adenoviral system in 293 A cells^3^. NRVMs are infected with adenovirus particles at MOI of 50. Ad-LacZ was used as a control. Small siRNA oligos (Sigma-Aldrich) were transfected with RNAiMAX reagent (Invitrogen) following manufacturer’s protocol. Total RNA was extracted 48 h later to determine the effectiveness of siRNA transfection.

### Patient samples and controls, hiPSC, and hiPSC-CM culture

Experiments with human cells/tissues are in compliance to the relevant ethical regulations. Human hypertrophic heart tissue samples and controls were described previously^3^. Tissue procurement was based on the receipt of written patient-informed consent and approved by the institutional review boards of Peking Union Medical College in China.

hiPSCs were reprogrammed from healthy human peripheral blood mononuclear cells with CytoTune-iPS Sendai Reprograming Kit (Thermo Fisher Scientific, A16518). The blood draw was from de-identified tissue repository of UT Southwestern, and its use is permitted by the Southwestern Human Research Protection Program without the need of formal IRB approval. The experiments have been performed under the oversight of the UT Southwestern Stem Cell Research Oversight (SCRO) Committee.

hiPSCs were cultured in mTeSR^TM^1 media (STEMCELL Technologies, 05850) and passaged approximately every 3–4 days (1:12-1:18 split ratio). iPSCs were induced to differentiate into cardiomyocytes, using previously described protocol^52^ with modifications. Briefly, hiPSCs were induced to differentiation to cardiomyocytes in CDM3 medium supplemented with WNT pathway activator CHIR99021 (4 μM) (Selleckchem, S2924) for 2–4 days, and then supplemented with 2 μM WNT inhibitor WNT-C59 (Selleckchem, S7037) for another 2 days. Media was then changed to RPMI 1640 with no glucose (Thermo Fisher Scientific, 11879020) supplemented with B27-supplement (Thermo Fisher Scientific, 17504044) for 10 days. On day 20, media was switched to cells maintaining media, RPMI 1640 medium, (Thermo Fisher Scientific, 11875093) supplemented with B27-supplement. Cardiomyocytes were used for experiments around 90 days after initiation of differentiation.

### Plasmid construction, reporter assays, and AAV9 transduction

Mouse *Timp1* promoter (-1205 to + 63) was amplified with Phusion enzyme (New England Biolabs) and subcloned into NheI-XhoI site of pGL3-basic vector. Reporter assay was done in 293 A cells^3^ with Luciferase Assay Substrate (Promega). Relative promoter activities were expressed as relative luminescence units normalized to co-transfected β-galactosidase activities in the cell. Three shRNAs (shTimp1-1: 5′-CACCCCTCTTGTTGCTATCACTGATCTCGAGATCAGTGATAGCAACAAGAGG-3′ shTimp1-2: 5′-CACCCCTGTTTATCTATCCCTTGCACTCGAGTGCAAGGGATAGATAAACAGG-3′ shTimp1-3: 5′-CACCCCACCTTATACCAGCGTTATACTCGAGTATAACGCTGGTATAAGGTGG-3′)​ corresponding to different regions of Timp1 were cloned into TRISPR2.0 donor plasmids and assembled into AAV9 TRISPR2.0 backbone plasmid using Golden Gate Assembly (New England Biolabs) following a published protocol^53^. In total, 2 × 10^12^ gc viruses/20 g of body weight in 200 μl of saline were i.v. injected via tail vein to mice 2 days before surgery.

### Statistical method

Data are presented as mean ± SEM. At least three independent experiments were performed for each assay. Statistical differences were calculated with two-tailed Student’s *t* test when comparing two conditions, and ANOVA was used when comparing more than two conditions. Changes were considered to be significant when *p* *<* 0.05.

## Electronic supplementary material

Supplementary Information

Description of Additional Supplementary Files

Supplementary Data 1

## Data Availability

All data including raw data are available upon request. Genomic data generated during the study are available in a public repository GEO (GSE120740, GSE120598). Original un-cropped western blots are provided in Supplementary Figure 7. The source data underlying Figs. 1b, 1d, 1e, 1f, 1g, 2b, 2c, 2d, 2e, 2g, 3b, 3c, 3d, 3e, 3f, 3g, 3h, 4d, 4e, 5a, 5b, 5c, 5d, 5e, 5f, 5g, 5i, 5j, 5k, 6b, 6c, 6d, 6e, 6f, 6i, 6j, 6k, 7b, 7c, 7e, and 8f and Supplementary Figs. 1c, 1d, 1f, 2d, 3b, 4b,4c, 5a, 5d, 5e, 5f, 6a, 6b, 6c, 6d, 6e, 6f, and 6g are provided in Supplementary Dataset 1.
