# Supplementary material for: Histone lysine dimethyl-demethylase KDM3A controls pathological cardiac hypertrophy and fibrosis
Source: Nat Commun. 2018 Dec 7;9:5230. doi: 10.1038/s41467-018-07173-2 (PMC6286331; doi:10.1038/s41467-018-07173-2)
Supplement: Supplementary file 1 — Supplementary Information [file 41467_2018_7173_MOESM1_ESM.pdf]

Supplementary figures.

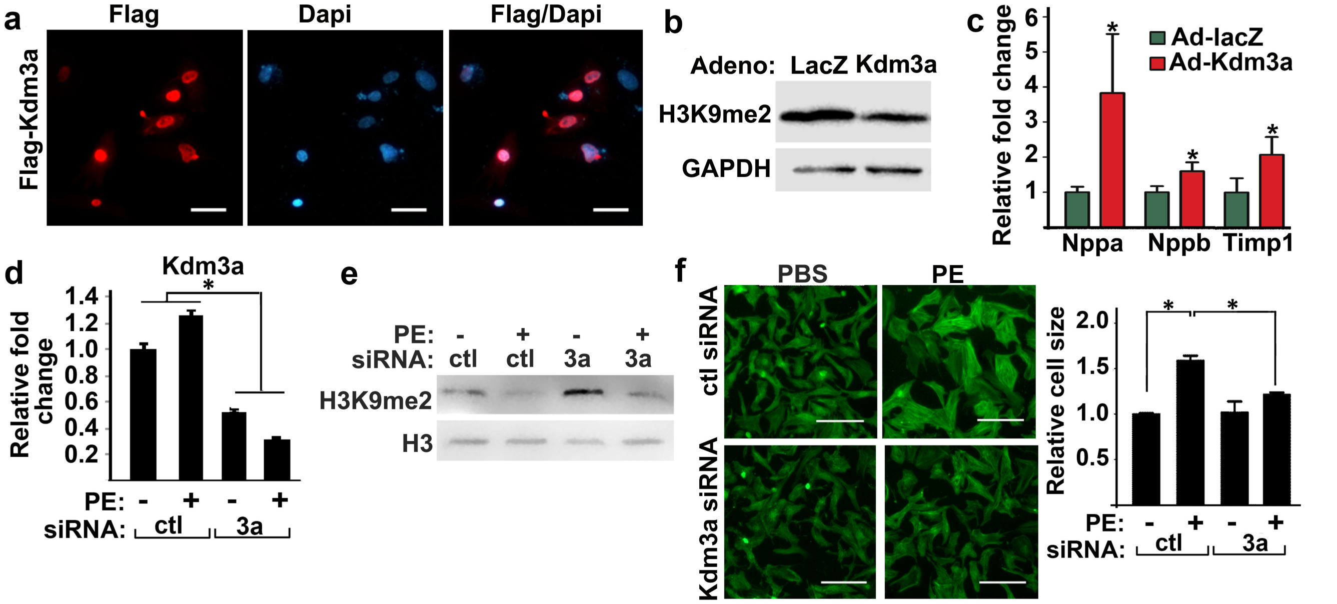

Supplementary Figure 1. KDM3A promotes cardiomyocyte hypertrophy. (a) Immunofluorescence staining of Flag-tagged KDM3A in NRVMs transduced with adenovirus expressing Flag-Kdm3a using anti-Flag antibody. Scale bar, 20 μM. (b) WB of H3K9me2 of NRVMS transduced with adenoviruses expressing LacZ (Ad-LacZ) or Kdm3a (Ad-Kdm3a). Gapdh was used as loading control. Overexpression of KDM3A in NRVMs resulted in downregulation of H3K9me2. (c) Relative mRNA of genes indicated from NRMVs transduced with either Ad-LacZ or Ad-Kdm3a. Overexpression of Kdm3a upregulated canonical genes associated with cardiomyocyte hypertrophy and Timp1. n=3±SEM, *, *p<0.05* (t test), relative to LacZ-transduced cells. (d-f) NRVMs were transfected with control or Kdm3a specific siRNA and stimulated with or without PE. Kdm3a knockdown (KD) resulted in approximately 50% downregulation of Kdm3a mRNA relative to control siRNA transfected cells (d). Kdm3a KD also resulted in upregulation of H3K9me2 (e). (f) Relative cell size of NRVMs in Kdm3a siRNA or control siRNA transfected cells and stimulated with or without PE. Kdm3a KD attenuated PE-stimulated myocyte hypertrophy *, *p<0.05* (ANOVA). Scale bar, 100 μm.

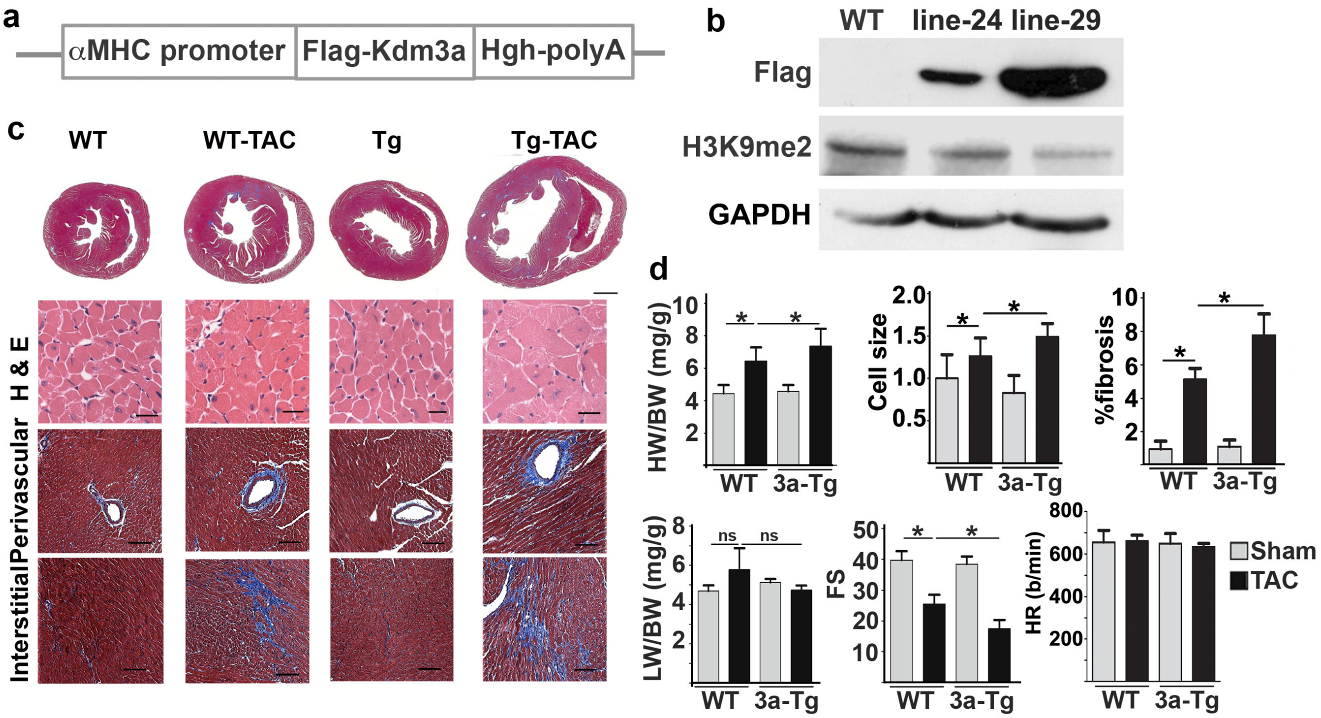

Supplementary Figure 2. KDM3A promotes TAC-induced hypertrophic remodeling. (a) Schematics of *Kdm3a* transgenic construct. (b) Western blot of expression levels of Flag-Kdm3a and H3K9me2 in the heart. GADPH was used as loading control. (c) H&E (top two panels) and trichrome (lower two panels) staining of histological sections of WT and *Kdm3a*-Tg (line 29) mouse hearts at week 6 post-sham and TAC surgery. Scale bars, 1mm (first panel), 20 μm (second panel), 100 μm (3rd and 4th panels). (d) HW/BW, LW/BW, FS, and Heart rate of *Kdm3a*-Tg (line 24) mouse hearts at week 6 post-sham and TAC surgery. n=5-9±SEM. **, p<0.05* (ANOVA).

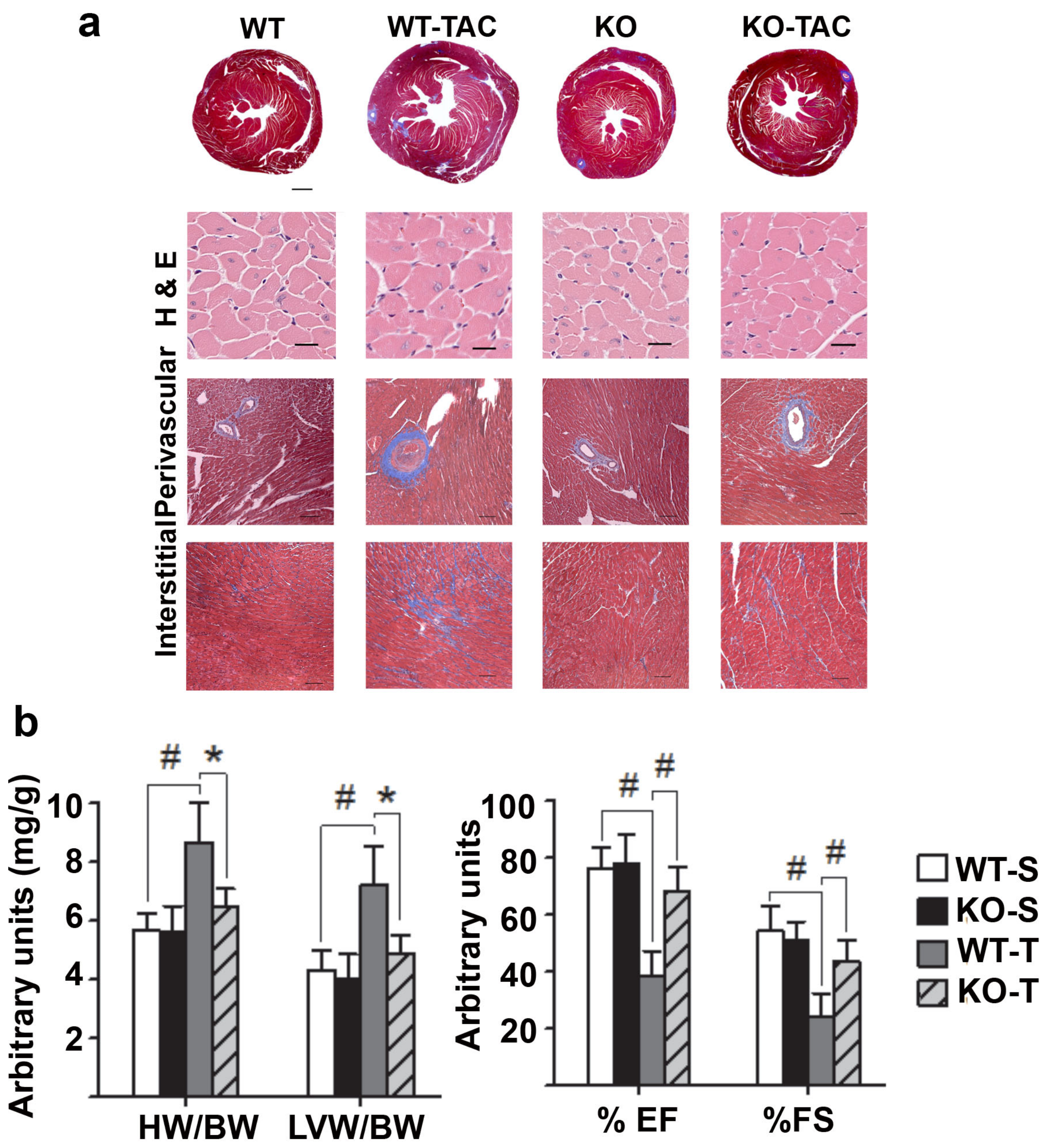

Supplementary Figure 3. *Kdm3a*-deficiency protects mice against hypertrophic remodeling in response to TAC injury. (a) H&E (top two panels) and trichrome (lower two panels) staining of histological sections of WT and *Kdm3a* KO mouse (in FVB/C57/Bl6 mixed background) hearts at week 6 post-sham and TAC surgery. Scale bars, 1mm (first panel), 20 μm (second panel), 100 μm (3rd and 4th panels). (b) HW/BW, LW/BW, ejection fraction (EF) and FS of *Kdm3a* KO mouse (in C57/Bl6 background) hearts at week 6 post-sham and TAC surgery. n=3-4±SEM. **, $p<0.05$. #, $p<0.01$* (ANOVA).

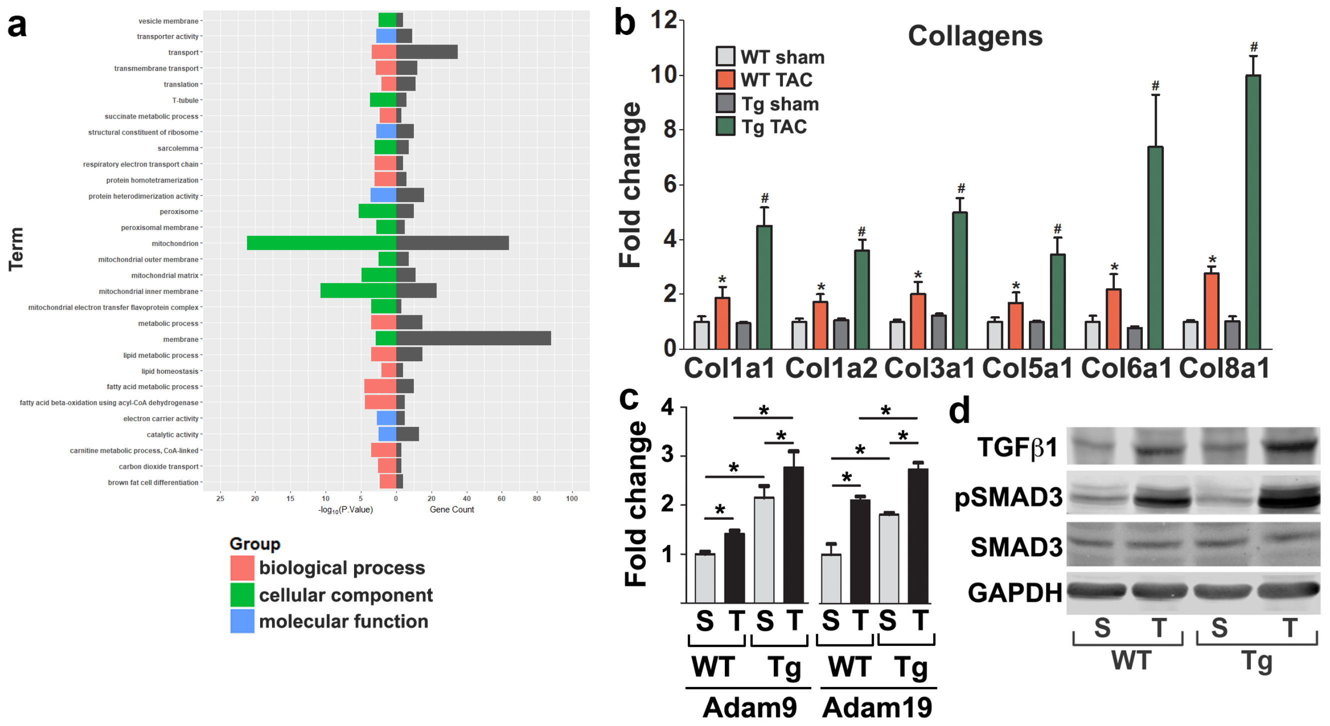

Supplementary Figure 4. (a) GO analysis of upregulated genes in *Kdm3a* KO TAC mouse hearts vs WT TAC littermates. (b) Relative fold change of mRNA of collagens in WT and Kdm3a-Tg mice at week 6 post-sham and TAC surgery. n=4±SEM. *, relative to sham, *p<0.05. #,* relative to WT TAC, *p<0.05* _(ANOVA). (c) Adam9 and Adam19 in WT and TG mouse heart at week 6 post-sham and TAC surgery. n=4±SEM, *,*p<0.05* (ANOVA). (d) Western blot of indicated proteins in WT and Kdm3a-Tg mouse hearts at week 6 post-sham and TAC surgery.

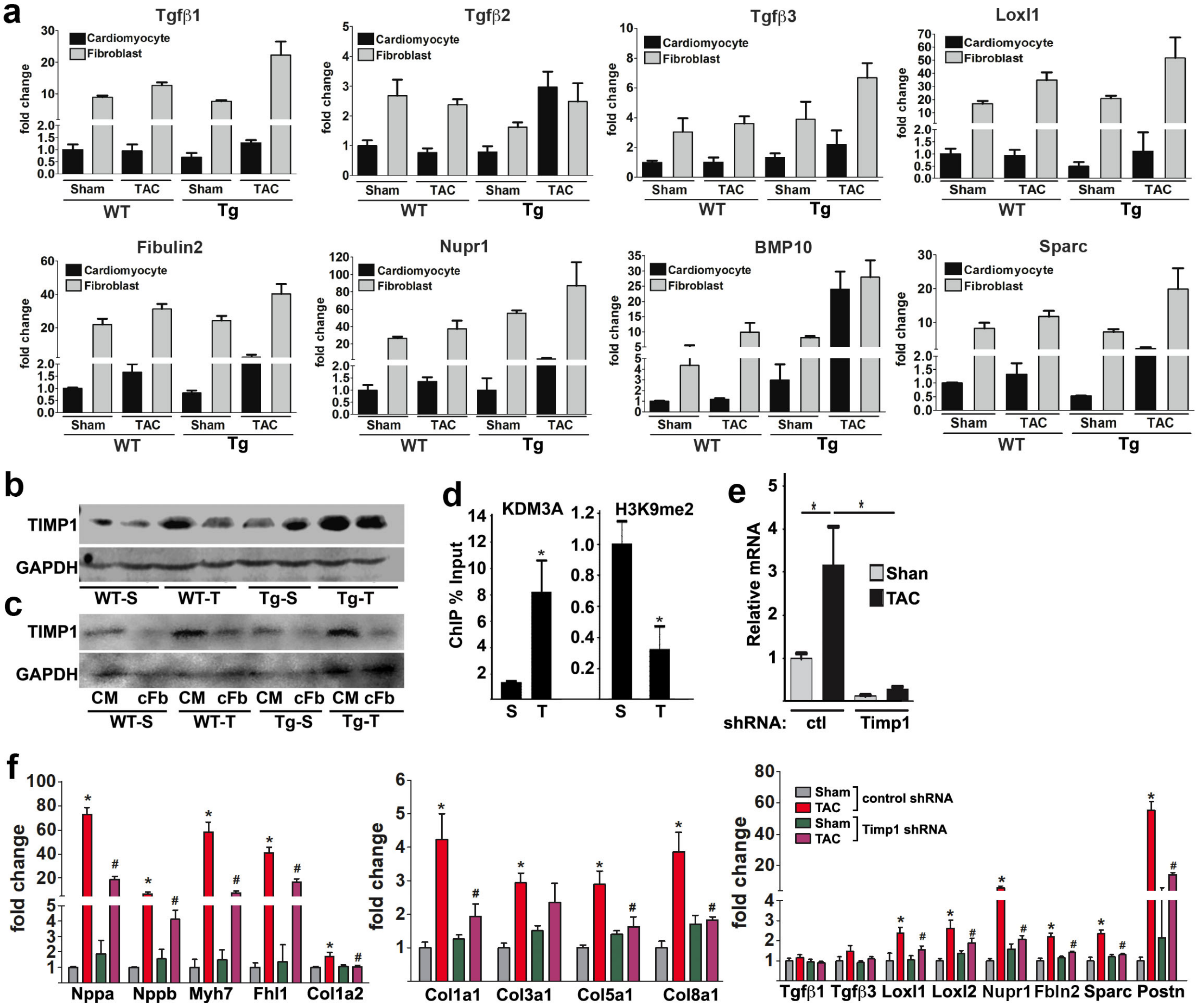

Supplementary Figure 5. (a) Relative fold change of transcripts of genes involved in fibrosis in cardiomyocytes (CM) and fibroblasts (cFb, non-cardiomyocytes) fractions of WT and Kdm3a-Tg mouse hearts at week 6 post-sham and TAC surgery n=3±SEM*, *,p<0.05* (ANOVA). (b) Western blot of TIMP1, showing upregulation of TIMP1 in *Kdm3a*-Tg mouse hearts and in response to TAC surgery. (c) Western blot of TIMP1 in CM and cFb isolated from WT and *Kdm3a*-Tg mouse at week 6 post-Sham and TAC surgery. (d) *Kdm3a*-Tg mouse hearts at weeks 6 post-Sham and TAC surgery were used for ChIP assay with antibodies against KDM3A (left panel) or H3K9me2 (right panel). The relative occupancies of KDM3A and H3K9me2 at Timp1 promoter were normalized against Input and expressed relative to Sham control. n=3±SEM*, *,p<0.05* (t test). (e) Relative fold change of Timp1 mRNA in Sham and TAC *Kdm3a*-Tg mouse hearts treated with control or Timp1 shRNA n=3±SEM*, *,p<0.05* (ANOVA). (f) Relative mRNA of hypertrophic and fibrotic genes in Sham and TAC *Kdm3a*-Tg mouse hearts treated with control or Timp1 shRNA. n=4±SEM. , * control shRNA TAC vs Sham, # Timp1 shRNA TAC vs control shRNA TAC. #, **,p<0.05* (ANOVA).

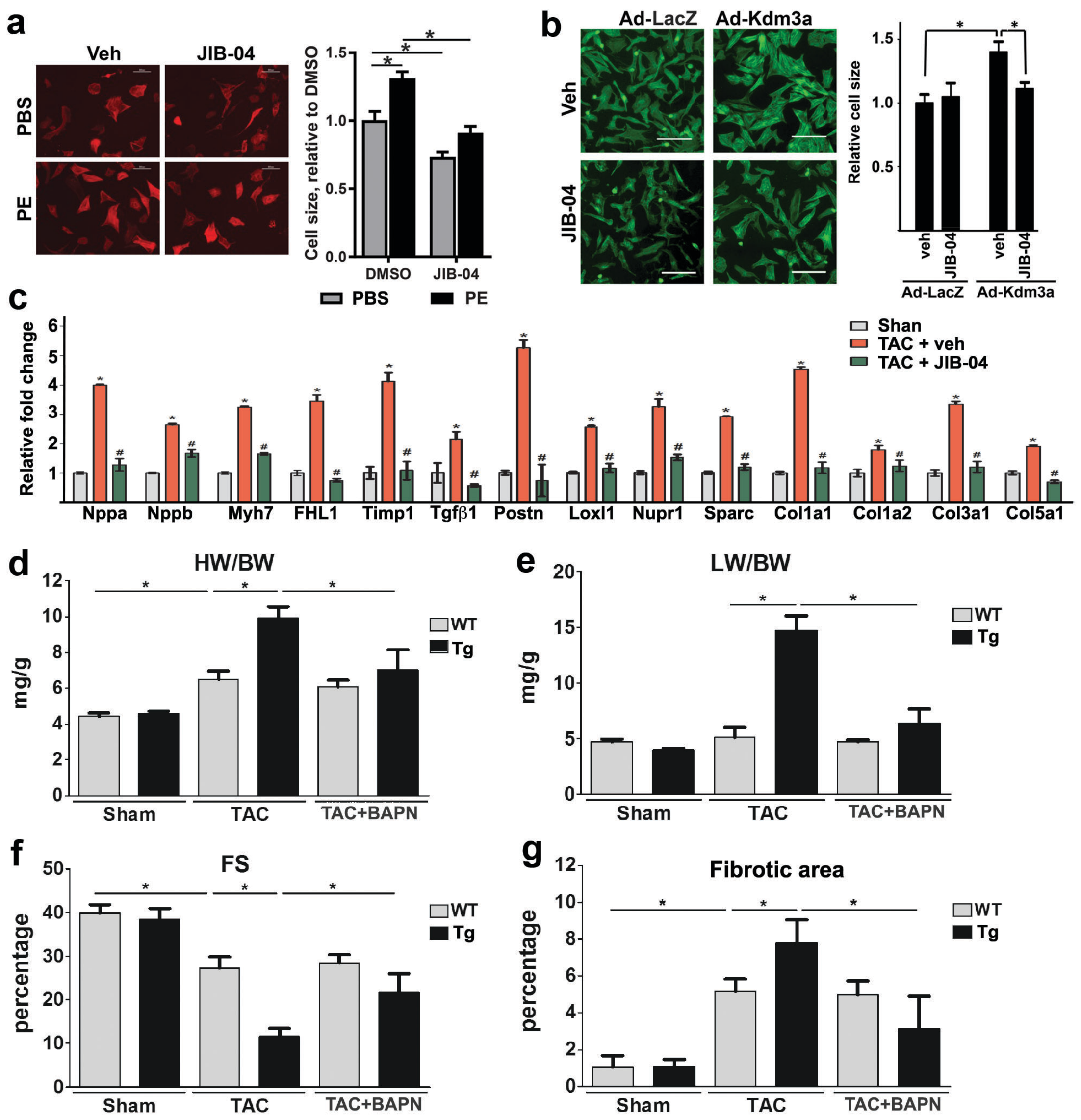

Supplementary Figure 6. (a) NRVMs were stimulated with PBS or PE, treated with JIB-04 or vehicle DMSO. JIB-04 suppressed PE-stimulated myocyte hypertrophy. n=3±SEM, *, *p<0.05* (ANOVA). (b) NRVMs transduced with either Ad-LacZ or Ad-Kdm3a were treated with JIB-04 or vehicle DMSO. JIB-04 suppressed Kdm3a-promoted myocyte hypertrophy n=3±SEM, *, *p<0.05* (ANOVA). Scale bar, 100 μm. (c) Relative mRNA of fetal genes and genes involved in fibrosis in *Kdm3a*-Tg sham and TAC mouse hearts treated with vehicle or JIB-04 starting at post-TAC week 3 for 4 weeks. mRNA transcripts were measured by qRT-PCR, normalized against internal Gapdh, and expressed relative to Sham mice. n=3±SEM. $^{*,\#}$ *p<0.05,* *TAC vs Sham, $^{\#}$JIB-04 vs vehicle n=3±SEM, *, *p<0.05* (ANOVA). (d-g) Kdm3a-Tg and WT littermates were subjected to TAC surgery and treated with Lox inhibitor β-aminoproprionitrile (BAPN) (8 mg/ml in drinking water). Echocardiograph was performed at week 6 post-TAC and hearts were harvested. HW/BW (c), LW/BW (d), FS (e), and relative fibrotic area (f) were measured. n=5-9±SEM, *, *p<0.05* (ANOVA).

Supplementary Figure 7 Full, un-cropped Western blots in main article figures and supplementary figures. Boxes indicate regions cropped in the main article.

Fig. 5h

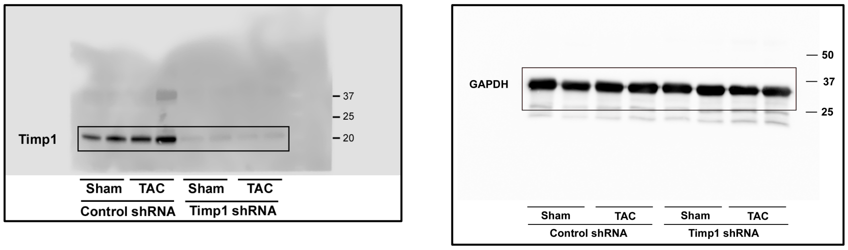

Fig. 6g

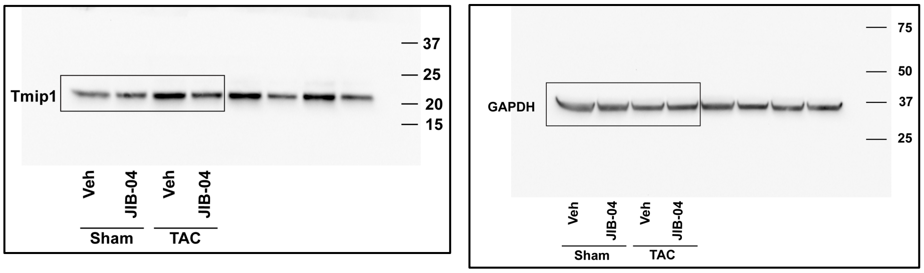

Fig. 8a

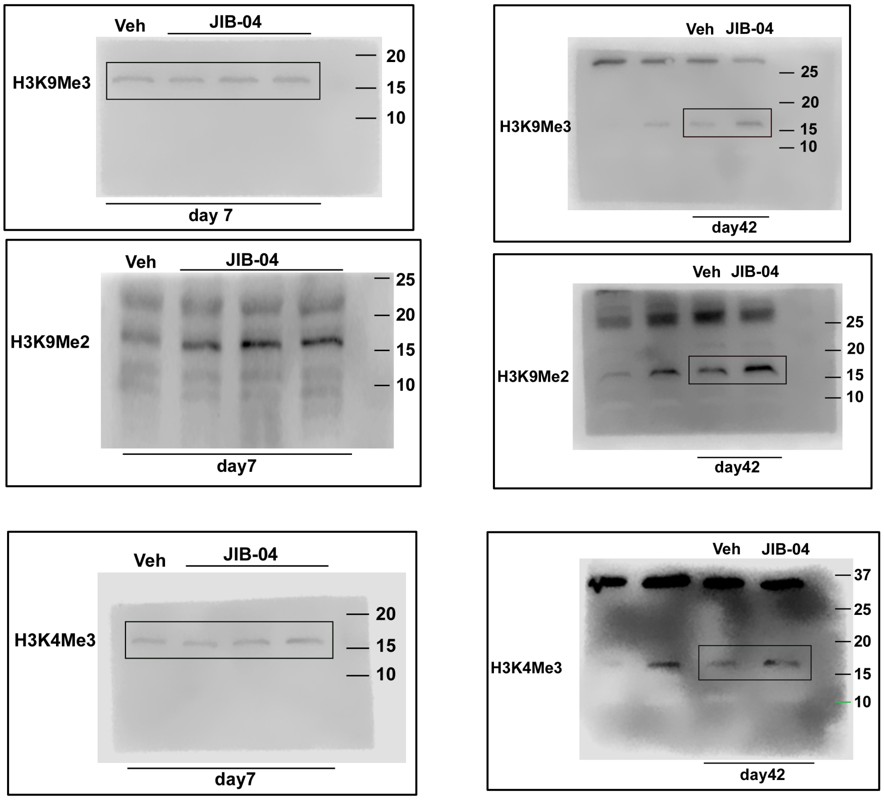

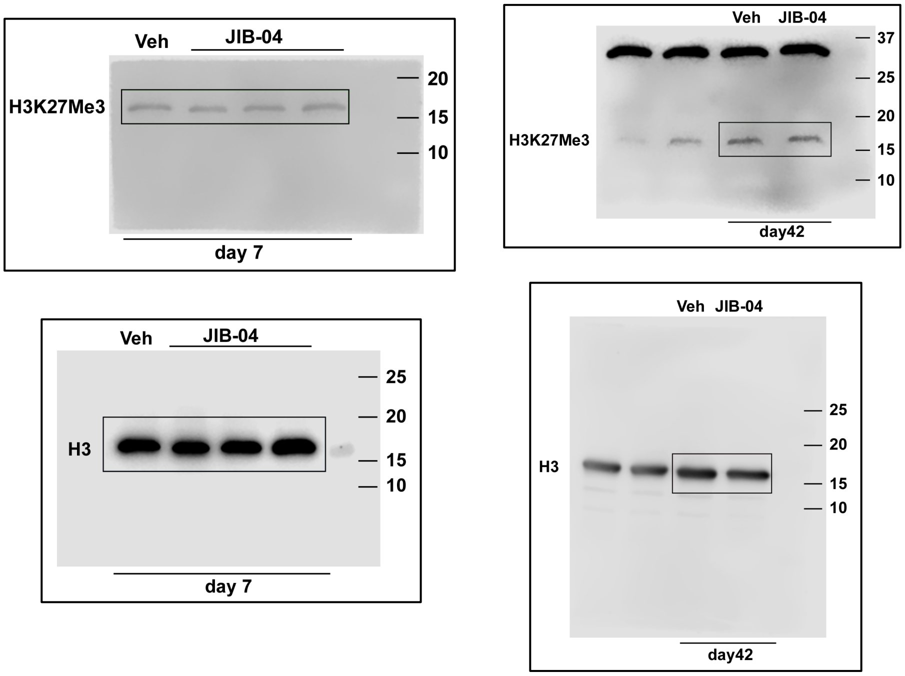
Veh
JIB-04
H3K27Me3
20
15
10
day 7
Veh JIB-04
37
25
20
15
10
H3K27Me3
day42
Veh
JIB-04
H3
25
20
15
10
day 7
Veh JIB-04
H3
25
20
15
10
day42

Fig. 8b

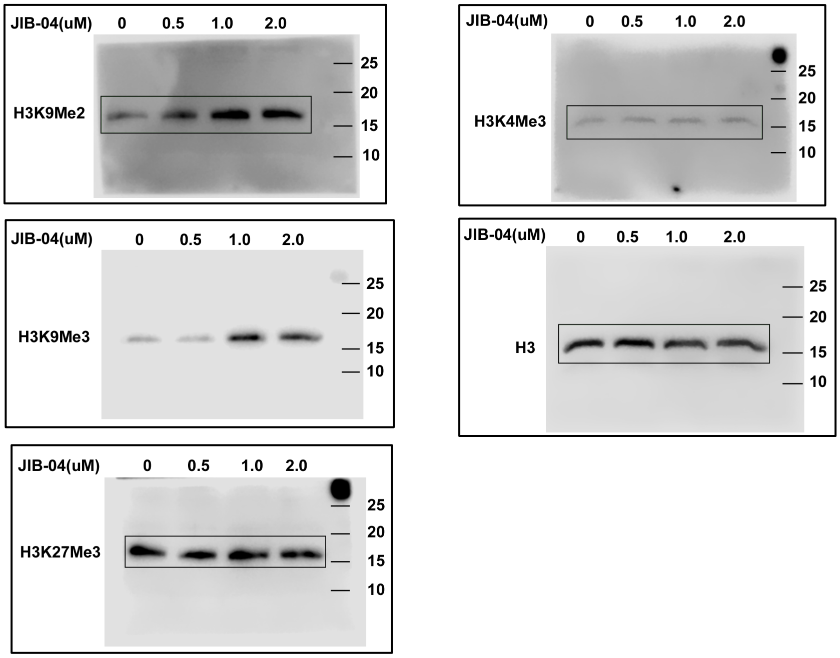
JIB-04(uM) 0 0.5 1.0 2.0
H3K9Me2
25
20
15
10
JIB-04(uM) 0 0.5 1.0 2.0
H3K4Me3
25
20
15
10
JIB-04(uM) 0 0.5 1.0 2.0
H3K9Me3
25
20
15
10
JIB-04(uM) 0 0.5 1.0 2.0
H3
25
20
15
10
JIB-04(uM) 0 0.5 1.0 2.0
H3K27Me3
25
20
15
10

Fig. 8c

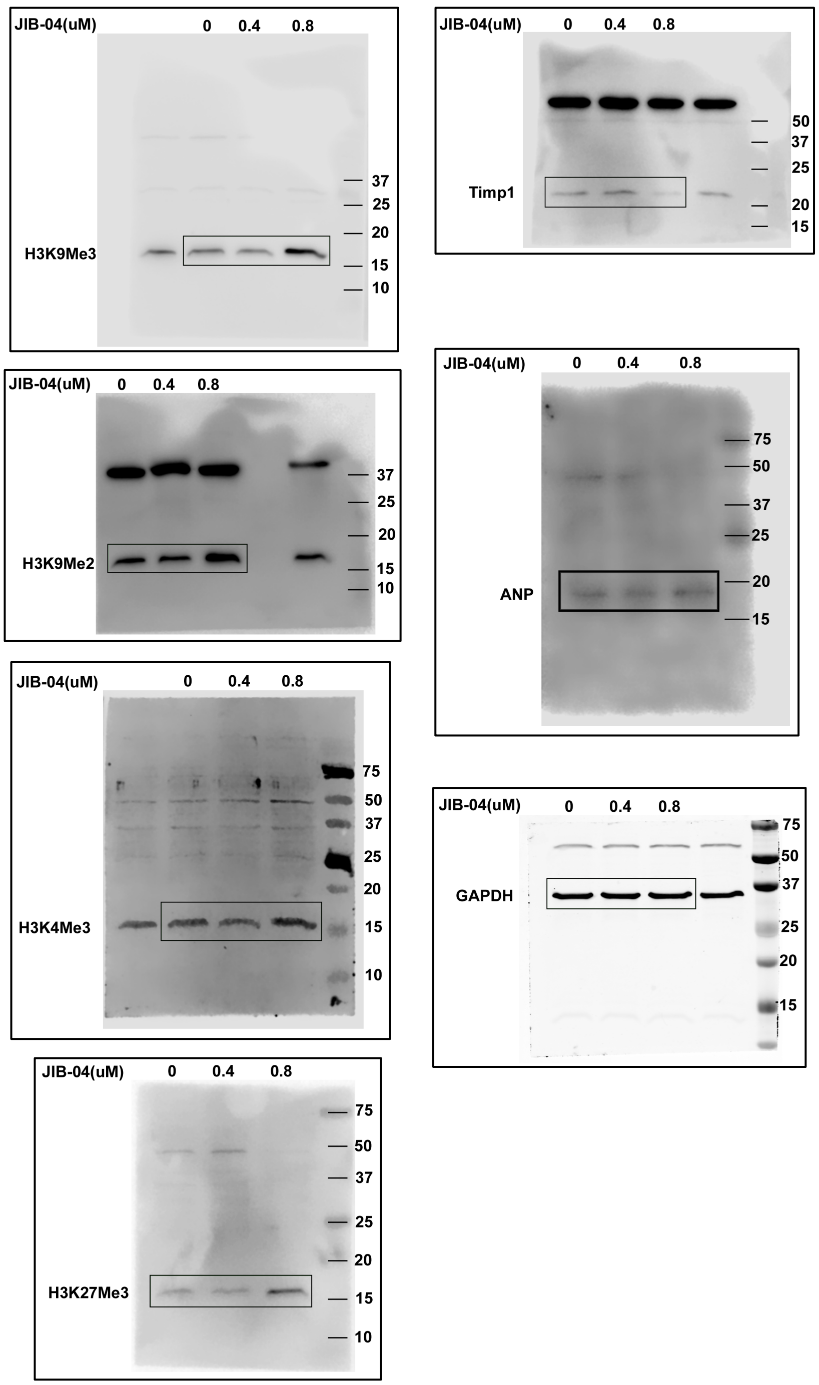
JIB-04(uM) 0 0.4 0.8
H3K9Me3
37
25
20
15
10
JIB-04(uM) 0 0.4 0.8
Timp1
50
37
25
20
15
JIB-04(uM) 0 0.4 0.8
H3K9Me2
37
25
20
15
10
JIB-04(uM) 0 0.4 0.8
ANP
75
50
37
25
20
15
JIB-04(uM) 0 0.4 0.8
H3K4Me3
75
50
37
25
20
15
10
JIB-04(uM) 0 0.4 0.8
GAPDH
75
50
37
25
20
15
JIB-04(uM) 0 0.4 0.8
H3K27Me3
75
50
37
25
20
15
10

Supplementary Figure 1b

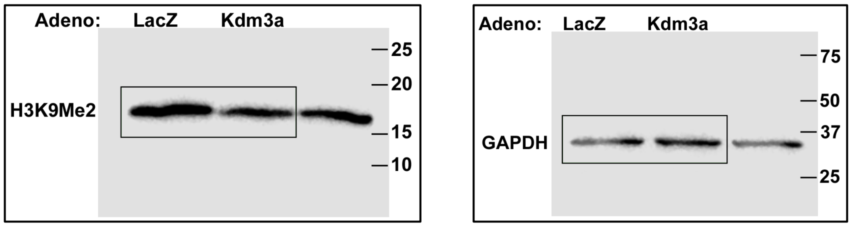

Supplementary Figure 1e

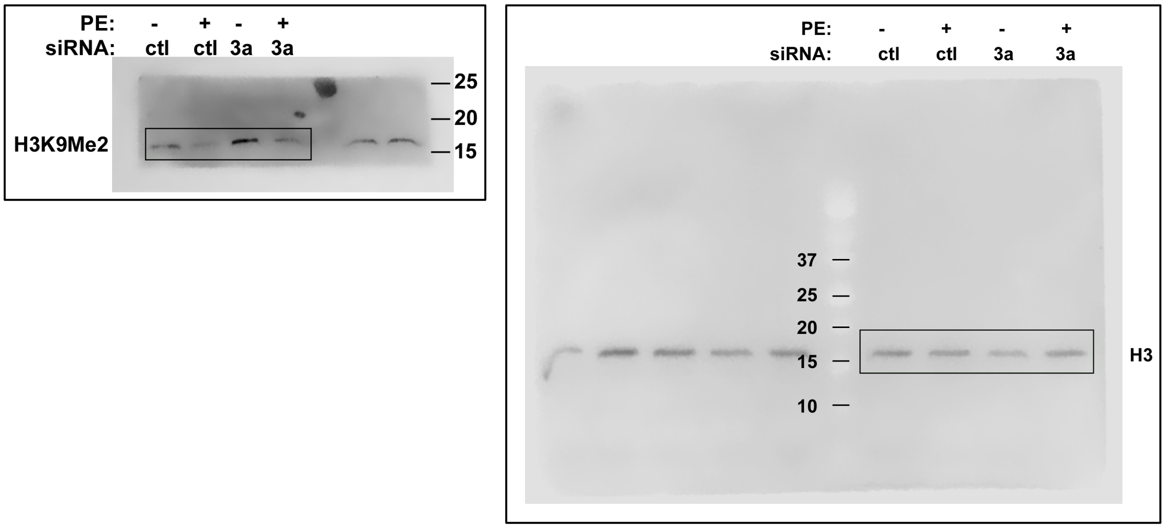

Supplementary Figure 2b

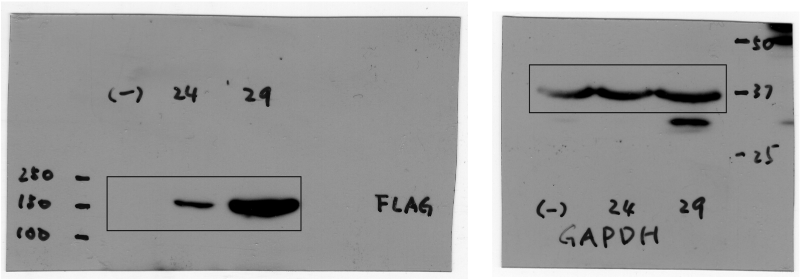

Supplementary Figure 4d

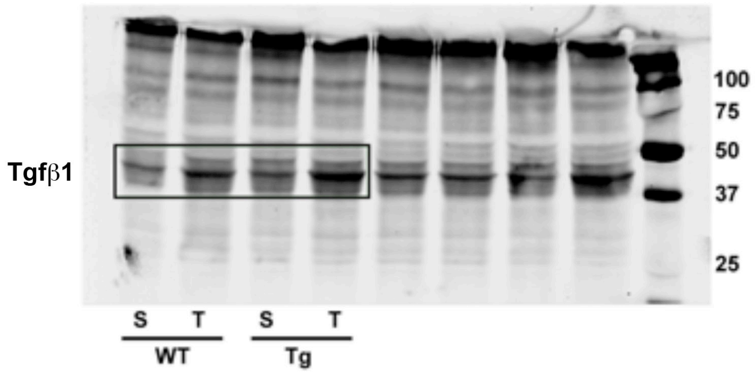

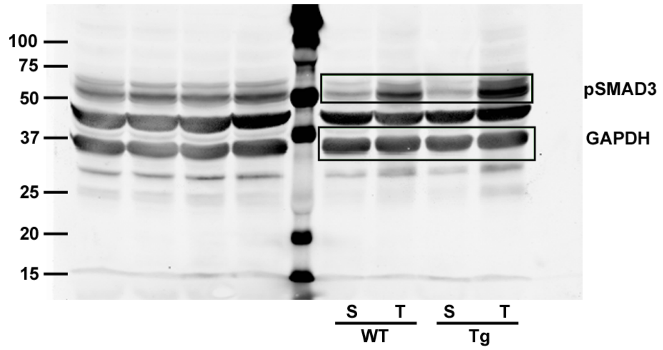

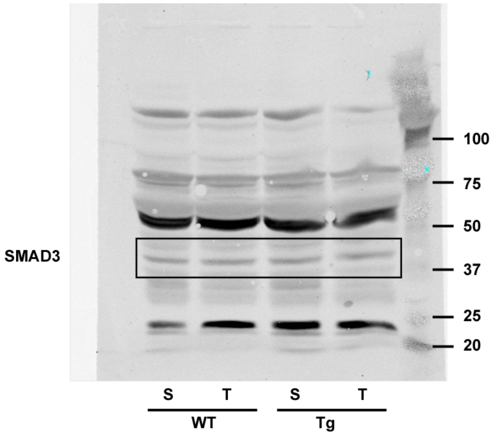

Supplementary Figure 5b

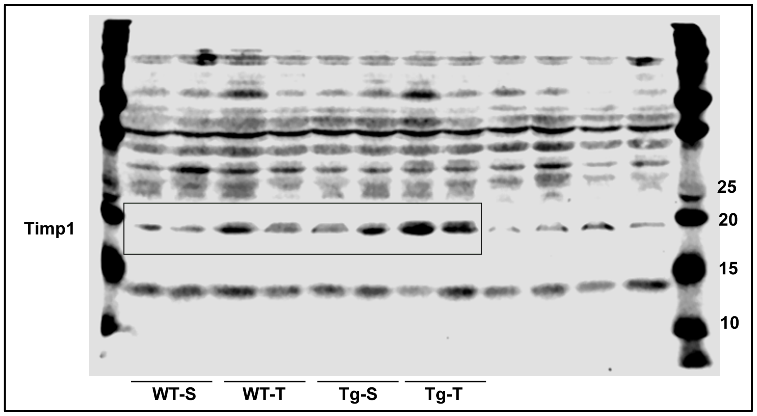

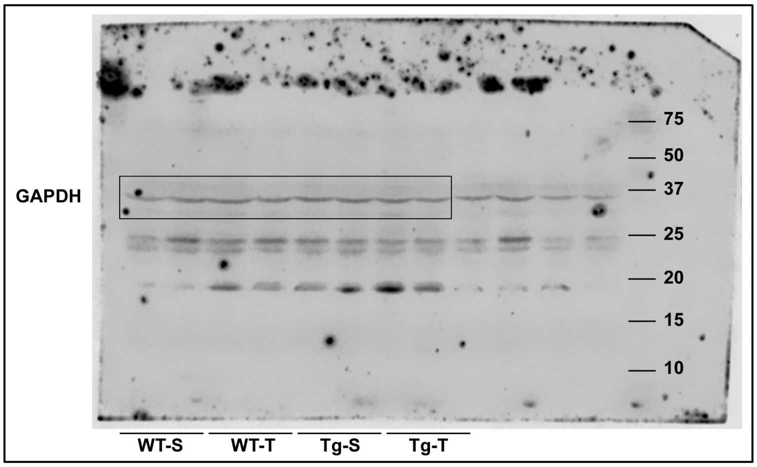

Supplementary Figure 5c

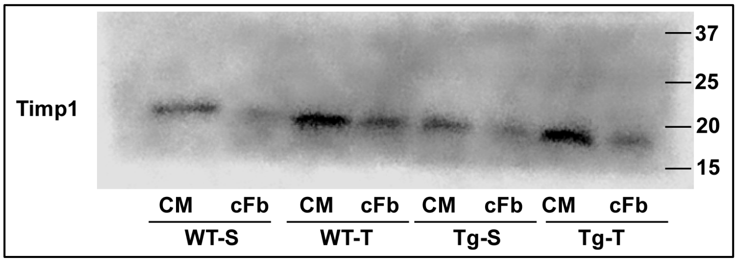

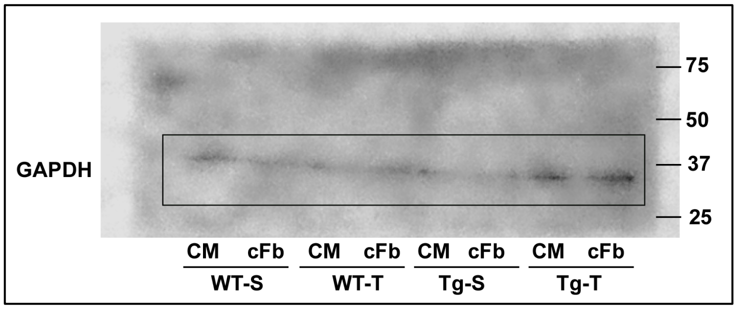

Supplementary table 1: primer sequences

Primer name	Sequence(5' to 3')
human GAPDH qPCR	p1: AGCCACATCGCTCAGACAC
	p2: GCCCAATACGACCAAATCC
human KDM3A qPCR	p1: CCAGCCTCAAAGGAAGACCT
	p2: ACTGCACCAAGAGTCGGTTT
mouse Gapdh qPCR	p1: GGCACAGTCAAGGCTGAGAATG
	p2: ATGGTGGTGAAGACGCCAGTA
mouse Nppa qPCR	p1: CAACACAGATCTGATGGATTTCA
	p2: CCTCATCTTCTACCGGCATC
mouse Nppb qPCR	p1: GTCAGTCGTTTGGGCTGTAAC
	p2: AGACCCAGGCAGAGTCAGAA
mouse Myh7 qPCR	p1: CGCATCAAGGAGCTCACC
	p2: CTGCAGCCGCAGTAGGTT
mouse Fhl1 qPCR	p1: GGCTTCTCAAAGACACTCAGG
	p2: TCGAACTTCTCCGACATGGT
mouse Tgfβ1 qPCR	p1: CTCCCGTGGCTTCTAGTGC
	p2: GCCTTAGTTTGGACAGGATCTG
mouse Tgfβ2 qPCR	p1: TCGACATGGATCAGTTTATGCG
	p2: CCCTGGTACTGTTGTAGATGGA
mouse Tgfβ3 qPCR	p1: CCTGGCCCTGCTGAACTTG
	p2: TTGATGTGGCCGAAGTCCAAC
mouse Timp1 qPCR	p1: AGCCTGGAGGCAGTGATTTC
	p2: GGGCCATCATGGTATCTCTGG
mouse LoxL1qPCR	p1: GAGTGCTATTGCGCTTCCC
	p2: GGTTGCCGAAGTCACAGGT
mouse LoxL2 qPCR	p1: ATTAACCCCAACTATGAAGTGCC
	p2: CTGTCTCCTCACTGAAGGCTC
mouse Nupr1 qPCR	p1: CCCTTCCCAGCAACCTCTAAA
	p2: TCTTGGTCCGACCTTTCCGA
mouse Fibulin2 qPCR	p1: CTGTGAAGACCAAGACGAGTG
	p2: CGTTGAGGATATAGCCCTCTGC
mouse Sparc qPCR	p1: GTGGAAATGGGAGAATTTGAGGA
	p2: CTCACACACCTTGCCATGTTT
mouse Postn qPCR	p1: CGGGAAGAACGAATCATTACA
	p2: ACCTTGGAGACCTCTTTTTGC
mouse Bmp10 qPCR	p1: ATGGGGTCTCTGGTTCTG
	p2: CAATACCATCTTGCTCCGTGAA
mouse Col1a1 qPCR	p1: GCTCCTCTTAGGGGCCACT
	p2: CCACGTCTCACCATTGGGG
mouse Col1a2 qPCR	p1: AGCCCTGGTTCTCGAGGT
	p2: CCGGTTGAACCACGATTG
mouse Col3a1 qPCR	p1: CTGTAACATGGAAACTGGGGAAA
	p2: CCATAGCTGAACTGAAAACCACC
mouse Col5a1 qPCR	p1: CTTCGCCGCTACTCCTGTTC
	p2: CCCTGAGGGCAAATTGTGAAAA
mouse Col6a1 qPCR	p1: AGGCCCTATTGGGCTTCAAG
	p2: GCCAGTGTATCCTCGCTCTC
mouse Col8a1 qPCR	p1: ACTCTGTCAGACTCATTCAGGC
	p2: CAAAGGCATGTGAGGGACTTG
mouse Adam9 qPCR	p1: GGAAGGCTCCCTACTCTCTGA
	p2: TCCAAAACTGGCATTCTCCAAA

mouse Adam19 qPCR	p1: TCAGTGGCGGACTTCAGAAAG
	p2: GCAAAAAGGTGCTCGTTCTTC
rat Gapdh qPCR	p1: ATCACCATCTTCCAGGAGCGA
	p2: AGCCTTCTCCATGGTGGTGAA
rat Nppa qPCR	p1: CACAGATCTGATGGATTTCAAGA
	p2: CCTCATCTTCTACCGGCATC
rat Nppb qPCR	p1: GTCAGTCGCTTGGGCTGT
	p2: CAGAGCTGGGGAAAGAAGAG
rat Fhl1 qPCR	p1: GGCTTCTCAAAGACACTCAGG
	p2: GTCGAACTTCTCAGACATGGTG
rat Myh6 qPCR	p1: TGCAGAAGAAACTGAAGGAAAA
	p2: GCTCCGCCTCTAGCTCCT
rat Timp1 qPCR	p1: CAGCAAAAGGCCTTCGTAAA
	p2: TGGCTGAACAGGGAAACACT
rat Kdm3a qPCR	p1: TTGCTCTGAGGTCTCTCCCAG
	p2: TGCTGTCTGTTGCTAGATGGG
Kdm3a-Tg mouse genotyping	p1: AGTGGTGGTGTAGGAAAGT
	p2: AACCACTGAGTAGATGGGTC
mouse Timp1 promoter	p1: AAAAAGCTAGCGCTGGCAGGAGGTTTTTGTG
	p2: AAAAACTCGAGAATCACTGCCTCCAGGCTTC
mouse Timp1 ChIP	p1: AGGAAGGACTGTGCATGACG
	p2: GGCCCCAGGATAAACCCAAA
