# Supplementary material for: Histone lysine dimethyl-demethylase KDM3A controls pathological cardiac hypertrophy and fibrosis
Source: Nat Commun. 2018 Dec 7;9:5230. doi: 10.1038/s41467-018-07173-2 (PMC6286331; doi:10.1038/s41467-018-07173-2)
Supplement: Supplementary file 2 — Description of Additional Supplementary Files [file 41467_2018_7173_MOESM2_ESM.docx]

**Description of Additional Supplementary Files**

**File Name: Supplementary Dataset:**

**Description:** Data file for the bar graphs.
